# Discrete cyclic systems and circle congruences

**DOI:** 10.1007/s10231-022-01219-5

**Published:** 2022-05-10

**Authors:** Udo Hertrich-Jeromin, Gudrun Szewieczek

**Affiliations:** TU Wien, Wiedner Hauptstraße 8-10/104, 1040 Vienna, Austria

**Keywords:** Discrete differential geometry, Lie sphere geometry, Möbius geometry, Orthogonal coordinate system, Cyclic system, Cyclic circle congruence, Normal line congruence, Dupin cyclide, Discrete flat front, 53A70 (Primary), 53A31, 53A35

## Abstract

We discuss integrable discretizations of 3-dimensional cyclic systems, that is, orthogonal coordinate systems with one family of circular coordinate lines. In particular, the underlying circle congruences are investigated in detail and characterized by the existence of a certain flat connection. Within the developed framework, discrete cyclic systems with a family of discrete flat fronts in hyperbolic space and discrete cyclic systems, where all coordinate surfaces are discrete Dupin cyclides, are investigated.

## Introduction

In [29], Ribaucour investigated circle congruences that admit a 1-parameter family of orthogonal surfaces and called those congruences *cyclic* (also called *normal*). The main motivations to study these special circle congruences seem to be twofold: firstly, a surface family orthogonal to a cyclic circle congruence gives rise to a special orthogonal coordinate system (*cyclic system*), where the orthogonal trajectories of one family are circular. As a consequence, two families of coordinate surfaces then consist of channel surfaces. Examples are provided by orthogonal systems with a family of surfaces that are parallel in a fixed space form (cf [24, 32]) and special cyclidic coordinate systems (also called totally cyclic), where all coordinate surfaces are Dupin cyclides [18, 32, 33]. Cyclic systems are widely used in physics and include rotational systems [28], as for example, spherical and toroidal coordinates.

Secondly, cyclic circle congruences can be employed to construct (families of) surfaces of various special types by imposing further (geometric) conditions on the cyclic circle congruence. Among them are, for example, pseudospherical surfaces related by Bianchi transformations [18, 20] or parallel families of flat fronts in hyperbolic space [11, 26]. The former example generalizes to a remarkable class of cyclic circle congruences given by curved flats in the space of circles (so-called flat spherical or hypercyclic systems) that come with an orthogonal family of Guichard surfaces [1]. Higher dimensional analogues lead to 3-dimensional conformally flat hypersurfaces [22] and, more generally, Möbius flat hypersurfaces [9, 14].

An integrable discretization of orthogonal coordinate systems was given in [4, 5, 7, 16] where those were introduced as higher-dimensional circular (principal) nets. The main goal of the present work is to explore discrete counterparts of cyclic circle congruences and their associated cyclic coordinate systems (see Def 11 and 16), based on this definition. In this way, we anticipate to pave the way for further studies in this context, inspired by the rich smooth theory as sketched above. For example, based on the observations in Sect. 4.2, we shall investigate relations of various approaches to flat fronts in hyperbolic space and, in particular, prove the existence of a Weierstrass-type representation for discrete flat fronts in [19]. Moreover, since the established theory naturally generalizes to higher-dimensional systems, it shall lead to discrete notions for 3-dimensional conformally flat hypersurfaces and Möbius flat hypersurfaces as orthogonal surfaces of discrete cyclic systems stemming from discrete flat fronts.

Another main goal of this paper is to further examine, and promote, the use of discrete connections that are given in the simplest way possible: by the “reflections” of the underlying ambient geometry. In our case, these will be Lie and M-Lie inversions (see Def 1) that can be used to generate cyclic circle congruences and their orthogonal nets in an efficient way. In a sometimes more implicit way, such connections have been used in the theory of discrete orthogonal systems for a long time, see [4, 5, 7, 8, 12, 13]. Here, we aim to present a more explicit treatment and, in particular, to explicitly investigate and employ the properties of connections built from Lie and M-Lie inversions in the context of cyclic systems. In this way, we hope to contribute to a methodologically systematic and transparent approach to the field.

The paper is structured as follows. After an introductory Sect. 2 on basic concepts and facts on circles and spheres that will be essential for what follows, the main notions of the text are presented in Sect. 3. Here we approach the discretization of smooth cyclic systems from two different angles: firstly, we consider discrete triply orthogonal systems that contain two coordinate surface families of discrete channel surfaces [23] and, therefore, have a family of circular orthogonal trajectories. In Sect. 3.2, we then take a different point of view: we start from a discrete 2-dimensional family of circles and investigate under what conditions there exists a family of orthogonal discrete Legendre maps that gives rise to a discrete cyclic system.

We then show that the proposed discretizations reflect properties that are well-known from the smooth theory, such as a constant cross-ratio of four orthogonal surfaces of a cyclic circle congruence, and the existence of discrete Ribaucour transformations between any two orthogonal surfaces of a discrete cyclic system (see Cor 19 and 20).

In Sect. 4, we pursue Ribaucour’s original approach and consider discrete cyclic circle congruences associated with a Ribaucour pair of discrete Legendre maps. In particular, we demonstrate that the circles that intersect the spheres of a Ribaucour sphere congruence in the point spheres of the two envelopes orthogonally constitute a discrete cyclic circle congruence (cf Thm 25).

As an application of the developed theory, we then investigate discrete cyclic circle congruences constructed from special discrete Ribaucour pairs, where at least one of the initial nets is totally umbilic. In this context, we will discuss parallel families of discrete flat fronts in hyperbolic space as described in [25, 31], and discrete cyclic systems where all coordinate surfaces are discrete Dupin cyclides (see § 4.2 and § 4.3).

The concepts used and discussed throughout this work, such as circles, orthogonal coordinate systems, are well defined in Möbius geometry but not in Lie sphere geometry. Nevertheless, we will work in a Lie sphere geometric setup, where we fix a Möbius geometry as subgeometry. This will enable us to use the elegant Lie sphere geometric descriptions for Dupin cyclides and discrete channel surfaces [23] that will play a key role in our investigations. Furthermore, enhancing methods developed in [30], this setup enables us to characterize discrete cyclic circle congruences by the existence of M-Lie inversions that interchange adjacent circles and induce a flat connection for the congruence (cf Thm 17). These maps then provide an efficient way to construct discrete Legendre maps orthogonal to the circle congruence and the other coordinate surfaces of the associated discrete cyclic systems.

## Preliminaries

Throughout this paper we shall work in a Möbius geometry, considered as a subgeometry of Lie sphere geometry. In this section we will sketch basic concepts of this setup and will formulate various facts on circles that will become useful later in the text. For more details or proofs the interested reader is referred to the exhaustive literature in this area; see, for example, the surveys [2, 15].

We shall exploit the hexaspherical coordinate model of Lie sphere geometry as introduced by Lie [27] and consider the 6-dimensional vector space ${\mathbb {R}}^{4,2}$ endowed with a metric of signature (4, 2). The *projective light cone* will be denoted by

$$\begin{aligned} {\mathbb {P}}({\mathcal {L}}): = \{ \text {span}\{ {\mathfrak {s}}\} \subset {\mathbb {R}}^{4,2} \ | \ \langle {\mathfrak {s}, \mathfrak {s}}\rangle =0 \} \subset {\mathbb {P}}({\mathbb {R}}^{4,2}) \end{aligned}$$

and represents the set of oriented 2-spheres. Two spheres $s_1$ and $s_2$ are in oriented contact if and only if any two corresponding vectors $\mathfrak {s}_1$ and $\mathfrak {s}_2$ in the light cone are orthogonal. Hence the set of contact elements, that is, of pencils of 2-spheres in oriented contact, is represented by the set of lines in the Lie quadric ${\mathbb {P}}({\mathcal {L}})$ or, equivalently, the subset of null 2-planes in ${\mathbb {R}}^{4,2}$ of the Grassmannian; it will be denoted by ${\mathcal{Z}}$

**Convention.** Throughout this work, homogeneous coordinates of elements in the projective space ${\mathbb {P}}({\mathbb {R}}^{4,2})$ will be denoted by the corresponding black letter; if statements hold for arbitrary homogeneous coordinates we will use this convention without explicitly mentioning it.

To obtain a Möbius geometry of oriented spheres as a subgeometry in this setup, we fix a point sphere complex $\mathfrak {p} \in {\mathbb {R}}^{4,2}$, $\langle {\mathfrak {p}, \mathfrak {p}}\rangle =-1$, and recover points, that is, spheres with radius zero, as elements in

$$\begin{aligned} {\mathbb {P}}({\mathcal {P}}) : = {\mathbb {P}}({\mathcal L}\cap \{\mathfrak {p}\}^\perp ). \end{aligned}$$

The group of Möbius transformations is then provided by all Lie sphere transformations that preserve the point sphere complex $\mathfrak {p}$. In particular, those preserve the (unoriented) angle $\varphi$ between two spheres $u,v \in {\mathbb {P}}({\mathcal {L}})$, given by

1 $$\begin{aligned} \cos \varphi = 1 - \frac{ \langle {\mathfrak {u},\mathfrak {v}}\rangle \langle {\mathfrak {p},\mathfrak {p}}\rangle }{ \langle {\mathfrak {u},\mathfrak {p}}\rangle \langle {\mathfrak {v},\mathfrak {p}}\rangle }. \end{aligned}$$

Furthermore, by choosing a vector $\mathfrak {q}\in {\mathbb {R}}^{4,2}\setminus \{ 0 \}$, $\langle {\mathfrak {p}, \mathfrak {q}}\rangle =0$, we distinguish a *quadric of constant curvature*

$$\begin{aligned} {\mathcal {Q}}: = \{ \mathfrak {n} \in {\mathbb {R}}^{4,2} \ | \ \langle {\mathfrak {n}, \mathfrak {n}}\rangle =0, \langle {\mathfrak {n}, \mathfrak {q}}\rangle =-1, \langle {\mathfrak {n}, \mathfrak {p}}\rangle =0 \}, \end{aligned}$$

with constant sectional curvature $-\langle {\mathfrak {q}, \mathfrak {q}}\rangle$, and obtain its complex of *hyperplanes*

$$\begin{aligned} {\mathcal {H}}: = \{ \mathfrak {n} \in {\mathbb {R}}^{4,2} \ | \ \langle {\mathfrak {n}, \mathfrak {n}}\rangle =0, \langle {\mathfrak {n}, \mathfrak {q}}\rangle =0, \langle {\mathfrak {n}, \mathfrak {p}}\rangle =-1 \}. \end{aligned}$$

In Lie sphere geometry, any element $a \in {\mathbb {P}}({\mathbb {R}}^{4,2})$ defines a *linear sphere complex* ${\mathbb {P}}({\mathcal {L}}\cap \{a\}^\perp )$, that is, a 3-dimensional family of 2-spheres. We distinguish three types of linear sphere complexes: if $\langle { \mathfrak {a}, \mathfrak {a}}\rangle = 0$, the complex is called *parabolic* and consists of all spheres that are in oriented contact with the sphere represented by *a*. If $\langle { \mathfrak {a}, \mathfrak {a}}\rangle < 0$, we say that the complex is *hyperbolic* and for $\langle { \mathfrak {a}, \mathfrak {a}}\rangle > 0$ we obtain an *elliptic* linear sphere complex.

In Möbius geometry, that is, for a fixed point sphere complex $\mathfrak {p}$, the latter has a beautiful geometric characterization: the linear sphere complex then contains all spheres that intersect the two spheres (that coincide up to orientation)

2 $$\begin{aligned} \mathfrak {s}_a^\pm \in \text {span}\{ {\mathfrak {a}, \mathfrak {p}}\} \end{aligned}$$

at the constant angle

$$\begin{aligned} \cos ^2 \varphi = \frac{K}{K-1}, \ \ \text {where } \ K = \frac{\langle {\mathfrak {a}, \mathfrak {p}}\rangle ^2}{\langle {\mathfrak {a}, \mathfrak {a}}\rangle \langle {\mathfrak {p}, \mathfrak {p}}\rangle }. \end{aligned}$$

In particular, the spheres in a linear sphere complex intersect the spheres $s_a^\pm$ orthogonally if and only if $\langle {\mathfrak {a}, \mathfrak {p}}\rangle =0$.

Conversely, suppose that $s \in {\mathbb {P}}({\mathcal {L}})$ is a sphere, $\mathfrak {s}\not \perp \mathfrak {p}$; then the elliptic linear sphere complex that contains all spheres intersecting *s* orthogonally is given by

3 $$\begin{aligned} \mathfrak {a} : = \mathfrak {s}+\langle {\mathfrak {s},\mathfrak {p}}\rangle \mathfrak {p}. \end{aligned}$$

Hence, a sphere $t \in {\mathbb {P}}({\mathcal {L}})$ intersects the sphere *s* orthogonally if and only if $\langle {\mathfrak {t}, \mathfrak {a}}\rangle =0$.

Any elliptic and hyperbolic linear sphere complex may be used to define a reflection: let $a \in {\mathbb {P}}({\mathbb {R}}^{4,2})$, $\langle { \mathfrak {a}, \mathfrak {a}}\rangle \ne 0$, then the *Lie inversion with respect to the linear sphere complex* ${\mathbb {P}}({\mathcal {L}}\cap \{a\}^\perp )$ is given by

$$\begin{aligned} \sigma _a:{\mathbb {R}}^{4,2} \rightarrow {\mathbb {R}}^{4,2}, \ \ \mathfrak {r} \mapsto \sigma _a(\mathfrak {r}) : = \mathfrak {r}-\frac{2\langle {\mathfrak {r}, \mathfrak {a}}\rangle }{\langle {\mathfrak {a}, \mathfrak {a}}\rangle }\mathfrak {a}. \end{aligned}$$

Any Lie inversion is an involution that maps spheres to spheres and preserves oriented contact between spheres. Moreover, we emphasize that a Lie inversion $\sigma _a$ preserves all elements that lie in the corresponding linear sphere complex ${\mathbb {P}}({\mathcal {L}}\cap \{a\}^\perp )$. In particular, Lie inversions that preserve the point sphere complex will play a crucial role:

### Definition 1

A Lie inversion $\sigma _a$ that preserves the point sphere complex, $\langle {\mathfrak {p}, \mathfrak {a}}\rangle =0$, will be called an *M-Lie inversion*.

Clearly, any M-Lie inversion is a Möbius transformation and generalizes the concept of Möbius inversions: if *a* determines an elliptic linear sphere complex, the M-Lie inversion becomes a Möbius inversion, that is, it provides a reflection in the spheres $s^\pm _a$ as given in (2). However, if the corresponding linear sphere complex is hyperbolic, it can be thought of as an antipodal map.

Note that the M-Lie inversion $\sigma _\mathfrak {p}$ with respect to the point sphere complex $\mathfrak {p}$ reverses the orientation of all spheres.

Let $s_1, s_2 \in {\mathbb {P}}({\mathcal {L}})$ be two spheres that are not in oriented contact and $\mathfrak {s}_1, \mathfrak {s}_2 \in {\mathcal {L}}$ fixed homogeneous coordinates, then the Lie inversion with respect to $\mathfrak {a}:=\mathfrak {s}_1-\mathfrak {s}_2$, interchanges the spheres $s_1$ and $s_2$. However, note that different choices for the homogeneous coordinates provide a 1-parameter family of Lie inversions. When $s_1$ and $s_2$ are not point spheres, $\mathfrak {s}_1,\mathfrak {s}_2\not \perp \mathfrak {p}$, then there is a unique M-Lie inversion, determined by

$$\begin{aligned} \mathfrak {a} : = \langle {\mathfrak {s}_2, \mathfrak {p}}\rangle \mathfrak {s}_1 - \langle {\mathfrak {s}_1, \mathfrak {p}}\rangle \mathfrak {s}_2, \end{aligned}$$

that maps $s_1$ to $s_2$ and preserves the point sphere complex $\mathfrak {p}$.

For later reference, we also recall [2, 30] that the formula for the cross-ratio of four spheres which are pairwise related by a Lie inversion simplifies: let $s_1, s_2 \in {\mathbb {P}}({\mathcal {L}})$, $\langle {\mathfrak {s}_1, \mathfrak {s}_2}\rangle \ne 0$, be two spheres that are not contained in the linear sphere complex ${\mathbb {P}}({\mathcal {L}}\cap \{a\}^\perp )$, then

4 $$\begin{aligned} \text {cr}(s_2, \sigma _a(s_2), \sigma _a(s_1), s_1) = 2\frac{\langle {\mathfrak {s}_1,\mathfrak {a}}\rangle \langle {\mathfrak {s}_2,\mathfrak {a}}\rangle }{\langle {\mathfrak {a},\mathfrak {a}}\rangle \langle {\mathfrak {s}_1,\mathfrak {s}_2}\rangle }. \end{aligned}$$

Using appropriate M-Lie inversions, this formula can also be used to compute the cross-ratio between four concircular point spheres.

### Circles in this framework

Throughout this text, we will consider unoriented circles, thus objects that belong to Möbius geometry. Hence, in the employed Lie geometric framework, we again fix a point sphere complex $\mathfrak {p}$ to distinguish a Möbius subgeometry. Then circles arise as special Dupin cyclides, where one family of curvature spheres are point spheres.

A *circle* $\Gamma$ is provided by an orthogonal splitting of ${\mathbb {R}}^{4,2}$,

$$\begin{aligned} \Gamma = (\gamma , \gamma ^\perp ) \in G_{(2,1)}^{{\mathcal {P}}} \times G_{(2,1)}, \end{aligned}$$

where $G_{(2,1)}^{{\mathcal {P}}}$ denotes the set of all (2, 1)-planes that are orthogonal to the point sphere complex $\mathfrak {p}$. Therefore, all spheres contained in $\gamma \in G_{(2,1)}^{{\mathcal {P}}}$ are point spheres, namely, the points of the circle. The spheres in $\gamma ^\perp$ are in oriented contact with all circle points, hence, provide a Möbius geometric pencil of spheres.

If we additionally fix a vector $\mathfrak {q} \in {\mathbb {R}}^{4,2}\setminus \{ 0 \}$, $\langle {\mathfrak {q}, \mathfrak {p}}\rangle =0$, to distinguish a space form ${\mathcal {Q}}$, we obtain *lines* in this space form as special circles satisfying $\mathfrak {q} \in \gamma$.

#### Fact 2

If two spheres $s_1, s_2 \in {\mathbb {P}}({\mathcal {L}})$ intersect (in the Möbius geometry given by $\mathfrak {p}$), then their circle of intersection is given by

$$\begin{aligned} \Gamma = (\gamma , \gamma ^\perp ) \in G_{(2,1)}^{{\mathcal {P}}} \times G_{(2,1)}, \ \text {where } \gamma ^\perp :=\text {span}\{ {\mathfrak {s}_1, \mathfrak {s}_2, \mathfrak {p}}\} . \end{aligned}$$

A crucial concept in the study of cyclic systems will be spheres and circles that intersect orthogonally. In what follows, we summarize several useful constructions in this realm and formulate them in the Lie sphere geometric framework.

Firstly, note that a circle $\Gamma =(\gamma , \gamma ^\perp )$ intersects a sphere $s \in {\mathbb {P}}({\mathcal {L}})$ orthogonally if and only if *s* intersects all spheres in $\gamma ^\perp$ orthogonally. In fact, a weaker condition is sufficient to ensure orthogonality:

#### Fact 3

Let $s_1, s_2 \in {\mathbb {P}}({\mathcal {L}})$ be two spheres that intersect in the circle $\Gamma = (\gamma , \gamma ^\perp )$. Then, $\Gamma$ intersects a sphere $t \in {\mathbb {P}}({\mathcal {L}})$ orthogonally if and only if the sphere *t* intersects $s_1$ and $s_2$ orthogonally.

#### Proof

Suppose that the sphere $t \in {\mathbb {P}}({\mathcal {L}})$ intersects $s_1$ and $s_2$ orthogonally, that is,

$$\begin{aligned} \langle {\mathfrak {t}, \mathfrak {s}_i + \langle {\mathfrak {s}_i, \mathfrak {p}}\rangle \mathfrak {p}}\rangle =0 \ \ \text {for } \ i=\{ 1,2 \}. \end{aligned}$$

Then, a straightforward computation shows that *t* intersects any sphere in

$$\begin{aligned} \gamma ^\perp = \text {span}\{ {\mathfrak {s}_1, \mathfrak {s}_2, \mathfrak {p}}\} \end{aligned}$$

orthogonally, which proves the claim. $\square$

**Fig. 1:**
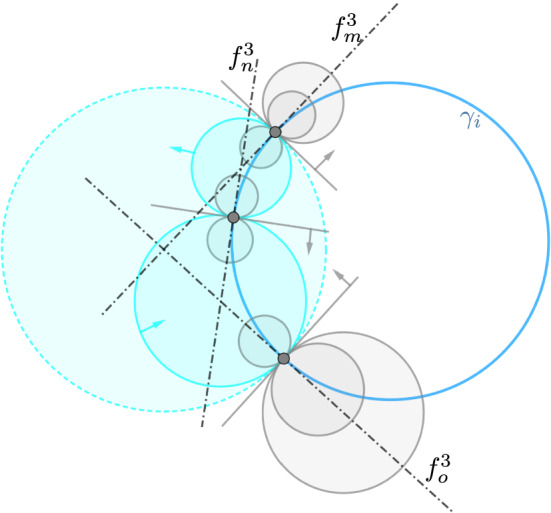
A circular orthogonal trajectory $\gamma _i$ of a discrete cyclic system with three orthogonal surfaces $f^3_m, f^3_n$ and $f^3_o$ that pairwise form (up to orientation) a discrete Ribaucour pair. The cyan spheres provide the spheres enveloped by two orthogonal surfaces

Spheres that are orthogonal to a fixed circle satisfy the following properties, which are also illustrated in Fig. 1.

#### Fact 4

The spheres that orthogonally intersect a circle $\Gamma$ in a fixed point $m\subset \gamma$ of the circle lie in two contact elements $m \in f_m, {\tilde{f}}_m \in {\mathcal {Z}}$ that coincide up to orientation, that is, ${\tilde{f}}_m = \sigma _{\mathfrak {p}}(f_m)$.

Moreover, for any two points $m,n\subset \gamma$ of the circle, the associated contact elements $f_m, {\tilde{f}}_m, f_n$ and ${\tilde{f}}_n$ of orthogonal spheres pairwise share a common sphere, that is,

$$\begin{aligned} f_m \cap f_n \ne \{ 0 \} \ \ \ \text { or } \ \ f_m \cap \sigma _{\mathfrak {p}}(f_n) \ne \{ 0 \}. \end{aligned}$$

#### Proof

Let $\Gamma = (\gamma , \gamma ^\perp )$ be a circle, given as the orthogonal intersection of two spheres $s_1,s_2\in {\mathbb {P}}({\mathcal {L}})$, and let $\mathfrak {m}\in \gamma$ represent a point of this circle. Without loss of generality, we choose homogeneous coordinates $\mathfrak {s}_i \in s_i$ such that $\langle {\mathfrak {s}_i, \mathfrak {p}}\rangle =-\langle {\mathfrak {s}_1, \mathfrak {s}_2}\rangle =1$ for $i =\{ 1, 2\}$.

Then, as a consequence of Fact 3, all spheres that intersect the circle $\Gamma$ in the point *m* orthogonally lie in the subspace

5 $$\begin{aligned} {\mathcal {O}}_m := \text {span}\{ {\mathfrak {s}_1 + \mathfrak {p}, \ \mathfrak {s}_2 + \mathfrak {p}, \ \mathfrak {m}}\} ^\perp . \end{aligned}$$

Since the subspace ${\mathcal {O}}_m^\perp$ has signature $(++0)$, we conclude that, for any point of the circle, the sought-after orthogonally intersecting spheres lie in two contact elements that coincide up to orientation.

Furthermore, since spheres that contain the points represented by $\mathfrak {m}, \mathfrak {n} \in \gamma$ and that are orthogonal to $\Gamma$ lie in the subspace $\text {span}\{ {\mathfrak {s}_1 + \mathfrak {p}, \ \mathfrak {s}_2 + \mathfrak {p}, \ \mathfrak {m}, \ \mathfrak {n}}\} ^\perp$, the second claim follows. $\square$

#### Fact 5

Let $\Gamma _1$ and $\Gamma _2$ be two circles and denote by $\mathfrak {s}_i, \mathfrak {t}_i \in \gamma _i^\perp \cap {\mathcal {L}}$, $i=\{1,2\}$, two spheres that determine the corresponding circles $\Gamma _i$. Then, the two circles lie on a common sphere if and only if the subspace

$$\begin{aligned} {\mathcal {S}} := \text {span}\{ {\mathfrak {s}_1, \mathfrak {s}_2, \mathfrak {t}_1, \mathfrak {t}_2, \mathfrak {p}}\} \subset {\mathbb {R}}^{4,2} \end{aligned}$$

is at most 4-dimensional.

#### Proof

Suppose that the two circles lie on the sphere $k \in {\mathbb {P}}({\mathcal {L}})$. Then, by Fact 2, we conclude that ${\mathcal {S}}$ is at most 4-dimensional.

To show the converse, we choose, without loss of generality, two spheres $\mathfrak {s}_1, \mathfrak {t}_1 \in \gamma _1^\perp \cap {\mathcal {L}}$ that intersect orthogonally and homogeneous coordinates such that $-\langle {\mathfrak {s}_1, \mathfrak {t}_1}\rangle =\langle {\mathfrak {s}_1, \mathfrak {p}}\rangle =\langle {\mathfrak {t}_1, \mathfrak {p}}\rangle =1$. If ${\mathcal {S}}$ is at most 4-dimensional, then there exist constants $\lambda _i \in {\mathbb {R}}$ such that

$$\begin{aligned} \lambda _1 \mathfrak {s}_1 + \lambda _2 \mathfrak {t}_1 + \lambda _3 \mathfrak {s}_2 + \lambda _4 \mathfrak {t}_2 + \mathfrak {p}=0. \end{aligned}$$

Thus, by setting $c^\pm := (\lambda _1+\lambda _2) \pm \sqrt{\lambda _1^2+\lambda _2^2}$ and $d^\pm :=1-c^\pm$, we obtain two spheres (with opposite orientation)

$$\begin{aligned} \lambda _1 \mathfrak {s}_1 + \lambda _2 \mathfrak {t}_1 + c^\pm \mathfrak {p} = -\lambda _3 \mathfrak {s}_2 - \lambda _4 \mathfrak {t}_2 - d^\pm \mathfrak {p} \end{aligned}$$

that lie in $\gamma _1^\perp \cap \gamma _2^\perp$ and, therefore, contain the two circles $\Gamma _1$ and $\Gamma _2$. This proves the claim. $\square$

#### Fact 6

Given a sphere $r \in {\mathbb {P}}({\mathcal {L}})$ and two point spheres $p_1, p_2 \in {\mathbb {P}}({\mathcal {P}})$ lying on it, the circle $\Gamma$ that intersects the sphere *r* orthogonally and passes through the points $p_1$ and $p_2$ is described by the (2, 1)-plane

6 $$\begin{aligned} \gamma : = \text {span}\{ {\mathfrak {p}_1, \ \mathfrak {p}_2, \ \mathfrak {r}+\langle {\mathfrak {r}, \mathfrak {p}}\rangle \mathfrak {p}}\} \in G^{{\mathcal {P}}}_{(2,1)}. \end{aligned}$$

#### Proof

Firstly, note that $\gamma$ is a (2, 1)-plane orthogonal to the point sphere complex $\mathfrak {p}$. Thus, $\gamma$ indeed describes a circle by $\Gamma := (\gamma , \gamma ^\perp ) \in G_{(2,1)}^{{\mathcal {P}}} \times G_{(2,1)}$. Furthermore, suppose that $\mathfrak {s} \in \gamma ^\perp \cap {\mathcal {L}}$ and consider the corresponding linear sphere complex with orthogonal intersection angle, that is,

$$\begin{aligned} \mathfrak {a} : = \mathfrak {s} + \langle {\mathfrak {s}, \mathfrak {p}}\rangle \mathfrak {p}. \end{aligned}$$

Then, because of $\langle {\mathfrak {s}, \mathfrak {r} + \langle {\mathfrak {r}, \mathfrak {p}}\rangle \mathfrak {p}}\rangle =0$, we conclude that

$$\begin{aligned} \langle {\mathfrak {r}, \mathfrak {a}}\rangle =0. \end{aligned}$$

Since this holds for any sphere in $\gamma ^\perp \cap {\mathcal {L}}$, the constructed circle $\Gamma$ intersects the sphere *r* orthogonally in the points $p_1$ and $p_2$. $\square$

As a consequence we obtain:

#### Fact 7

Given a sphere $s \in {\mathbb {P}}({\mathcal {L}})$ and two circles $\Gamma _1$ and $\Gamma _2$ that intersect the sphere orthogonally in the points $p_1^1, p_2^1$ and $p_1^2, p_2^2$. Then the circles lie on a common sphere if and only if the four points are concircular, that is, any homogeneous coordinate vectors $\mathfrak {p}_i^j$ are linearly dependent.

#### Proof

The sought-after sphere has to lie in $\gamma _1^\perp \oplus \gamma _2^\perp$, where $\gamma _i$ is given by (6). Thus, the subspace $\gamma _1 \oplus \gamma _2$ has to be a 4-dimensional space. $\square$

### Ribaucour transformations between two circles

Recall [10, 23] that any two cospherical circles are related by a Ribaucour transformation, that is, they envelop a common circle congruence. For two unparametrized circles on a sphere, there are at most two Ribaucour transformations that induce different Ribaucour correspondences between the point spheres of the two circles.

Those point-to-point mappings can be described by two M-Lie inversions:

#### Lemma 8

Let $\Gamma _1$ and $\Gamma _2$ be two circles that are not tangent to each other and lie on the sphere $k \in {\mathbb {P}}({\mathcal {L}})$. Then the two Ribaucour correspondences between $\Gamma _1$ and $\Gamma _2$ are induced by the two M-Lie inversions $\sigma _a$ and $\sigma _{{\tilde{a}}}$ determined by the linear sphere complexes

$$\begin{aligned} \mathfrak {a} : = \langle {\mathfrak {s}_2, \mathfrak {p}}\rangle \mathfrak {s}_1 - \langle {\mathfrak {s}_1, \mathfrak {p}}\rangle \mathfrak {s}_2 \ \ \ \text {and } \ \ \tilde{\mathfrak {a}} : = \langle {\sigma _\mathfrak {p}(\mathfrak {s}_2), \mathfrak {p}}\rangle \mathfrak {s}_1 - \langle {\mathfrak {s}_1, \mathfrak {p}}\rangle \sigma _\mathfrak {p}(\mathfrak {s}_2), \end{aligned}$$

where $\mathfrak {s}_i \in \gamma _i^\perp$, $i= \{ 1,2 \}$, are two spheres that intersect *k* orthogonally.

We remark that, for two touching circles $\Gamma _1$ and $\Gamma _2$ on a common sphere $k \in {\mathbb {P}}({\mathcal {L}})$, there exists only one Ribaucour correspondence: in this case, either $\langle {\mathfrak {s}_1, \mathfrak {s}_2}\rangle =0$ or $\langle {\mathfrak {s}_1, \sigma _\mathfrak {p}(\mathfrak {s}_2)}\rangle =0$ and, therefore, one of the M-Lie inversions described in Lemma 8 degenerates. Thus, between two touching circles we obtain a unique Ribaucour correspondence.

#### Proof

Without loss of generality, we assume that

$$\begin{aligned} \langle {\mathfrak {s}_1, \mathfrak {p}}\rangle =\langle {\mathfrak {s}_2, \mathfrak {p}}\rangle =\langle {\mathfrak {k}, \mathfrak {p}}\rangle =1. \end{aligned}$$

Then, since $s_1$ and $s_2$ intersect the sphere *k* orthogonally, from (1) we conclude that

$$\begin{aligned} \langle {\mathfrak {s}_1, \mathfrak {k}}\rangle =\langle {\mathfrak {s}_2, \mathfrak {k}}\rangle =-1. \end{aligned}$$

Hence, the Lie inversions $\sigma _a$ and $\sigma _{{\tilde{a}}}$ preserve the sphere *k*, as well as the point sphere complex $\mathfrak {p}$. Furthermore, they map $s_1$ to $s_2$ and $\text {span}\{ {\sigma _\mathfrak {p}(\mathfrak {s}_2)}\}$, respectively. Therefore, they are M-Lie inversions and interchange point spheres of the circles $\Gamma _1$ and $\Gamma _2$ with each other.

For any induced pair of point spheres $p_1$ and $p_2$, there exists a sphere $t \in {\mathbb {P}}({\mathcal {L}})$ that contains these points and is in oriented contact with $s_1$ and $s_2$. Then, *t* is preserved by those M-Lie inversions and intersects *k* orthogonally.

Moreover, the (2, 1)-plane $\text {span}\{ {\mathfrak {t}, \mathfrak {k}, \mathfrak {p}}\}$ determines a circle that is tangent to the circles $\Gamma _1$ and $\Gamma _2$ at the points $p_1$ and $p_2$. This proves the claim. $\square$

The generic ambiguity of the Ribaucour correspondence between two cospherical circles is eliminated by the choice of one admissible point sphere pair:

#### Corollary 9

Let $\Gamma _1$ and $\Gamma _2$ be two cospherical circles and let $\mathfrak {p}_i \in \gamma _i \cap {\mathcal {L}}$, $i=\{1,2\}$, represent a pair of points that is contained in a circle tangent to $\Gamma _1$ and $\Gamma _2$. Then, there exists a unique Ribaucour transformation between the circles that extends the correspondence between the points $p_1$ and $p_2$.

#### Proof

Let $\Gamma _1$ and $\Gamma _2$ be two circles lying on the sphere $k \in {\mathbb {P}}({\mathcal {L}})$. Moreover, we denote by $\mathfrak {s}_1 \in \gamma _1^\perp \cap {\mathcal {L}}$ a sphere that is orthogonal to *k* and define the sphere

$$\begin{aligned} \mathfrak {s}_{12} : = \langle {\mathfrak {p}_1, \mathfrak {p}_2}\rangle \mathfrak {s}_1 - \langle {\mathfrak {s}_1, \mathfrak {p}_2}\rangle \mathfrak {p}_1. \end{aligned}$$

The sphere $s_{12}$ contains the point pair $(p_1, p_2)$ and lies in the contact element $\text {span}\{ {s_1, p_1}\}$. Then the sphere $\mathfrak {s}_2 \in \gamma _2^\perp \cap \text {span}\{ {\mathfrak {s}_{12}, \mathfrak {p}_2}\}$ provides the construction for the sought-after Ribaucour correspondence: the M-Lie inversion determined by the linear sphere complex

$$\begin{aligned} \mathfrak {a} : = \langle {\mathfrak {s}_2, \mathfrak {p}}\rangle \mathfrak {s}_1 - \langle {\mathfrak {s}_1, \mathfrak {p}}\rangle \mathfrak {s}_2 \end{aligned}$$

induces the Ribaucour correspondence that maps $p_1$ onto $p_2$. $\square$

Conversely, if two circles $\Gamma _1$ and $\Gamma _2$ are related by an M-Lie inversion $\sigma$, then $\sigma$ induces a smooth Ribaucour transformation between the two circles: let

$$\begin{aligned} t \mapsto (\mathfrak {c}_1(t), \ \sigma _a(\mathfrak {c}_1(t)) \end{aligned}$$

be a simultaneous parametrization of the two circles, where $\mathfrak {c}_1(t) \in \gamma _1 \cap {\mathcal {L}}$. For any sphere $\mathfrak {s}_1 \in \gamma _1^\perp$, the map

$$\begin{aligned} t \mapsto \mathfrak {s}(t) : = \text {span}\{ {\mathfrak {s}_1, \mathfrak {c}_1(t)}\} \cap \text {span}\{ {\sigma _a(\mathfrak {s}_1), \sigma _a(\mathfrak {c}_1(t)}\} \cap {\mathcal {L}} \end{aligned}$$

defines a 1-parameter family of spheres that is in oriented contact with both circles. Hence, by [23, Thm 2.15], we conclude that $t \mapsto s(t)$ gives rise to a Dupin cyclide with curvature circles $\Gamma _1$ and $\Gamma _2$, which proves the claim.

## Discrete cyclic systems

In this section, we develop the main notions of this paper from two different points of view: firstly, we consider discrete cyclic systems, that is, discrete orthogonal coordinate system that have two families of discrete channel surfaces as coordinate surfaces. Secondly, we investigate under which conditions a discrete circle congruence is cyclic, thus is the underlying circle congruence of a discrete cyclic system.

**Notation.** Throughout the text, we will adopt the following notation conventions for domains of discrete maps: we will consider a simply connected subset of the lattice ${\mathbb {Z}}^3$, organized into vertices $\bar{{\mathcal {V}}}$, edges $\bar{{\mathcal {E}}}$, and faces $\bar{{\mathcal {F}}}$; for a 2-dimensional “slice,” modeled in ${\mathbb {Z}}^2$, we will use ${\mathcal {V}}$, ${\mathcal {E}}$, and ${\mathcal {F}}$, for the sets of vertices, edges, and faces, respectively. Thus our domains will be (rather trivial) quadrilateral or cubical cell complexes, where only cells of the dimensions 0, 1, and 2 will play a role. Furthermore, $I\subset {\mathbb {Z}}$ will denote (the vertex set of) a discrete (closed) interval.

The domains under consideration are assumed to be sufficiently large, that is, in each coordinate direction there exist at least three vertices.

### Definition and basic properties

In the spirit of [4, 7], we consider discrete triply orthogonal systems as principal contact element nets:

#### Definition 10

A *discrete triply orthogonal system* is a map

$$\begin{aligned} f:\bar{{\mathcal {V}}} \rightarrow {\mathcal {Z}}\times {\mathcal {Z}} \times {\mathcal {Z}}, \ i \mapsto f_i= (f_i^1,f_i^2,f_i^3) \end{aligned}$$

such that

(i) at each vertex the point spheres of the contact elements coincide,
  $$\begin{aligned} f_i^1 \cap f_i^2 \cap f_i^3 =: f_i^p \in {\mathbb {P}}({\mathcal {P}}), \end{aligned}$$
(ii) at each vertex the spheres of different contact elements intersect orthogonally and
(iii) any two adjacent contact elements of the same family intersect,
  $$\begin{aligned} f_i^\mu \cap f_j^\mu =: s_{ij}^\mu \in {\mathbb {P}}({\mathcal {L}}), \ \mu =\{ 1,2,3 \}. \end{aligned}$$

The map $f^p: {\bar{{\mathcal {V}}}} \rightarrow {\mathbb {P}}({\mathcal {P}})$ will be called the *point sphere map* of *f* and provides a 3-dimensional circular net.

We remark that a map $f:\bar{{\mathcal {V}}} \rightarrow {\mathcal {Z}}\times {\mathcal {Z}} \times {\mathcal {Z}}$ satisfies conditions (i) and (ii) if and only if, at any vertex $i\in \bar{{\mathcal {V}}}$, any two spheres $s_i^\lambda$ and $s_i^\mu$ of any two different contact elements $f_i^\lambda$ and $f_i^\mu$ fulfill

$$\begin{aligned} \langle {\mathfrak {s}_i^\lambda , \mathfrak {s}_i^\mu + \langle {\mathfrak {p}, \mathfrak {s}_i^\mu }\rangle \mathfrak {p}}\rangle =0. \end{aligned}$$

Note that this condition is symmetric in $\lambda ,\mu \in \{1,2,3\}$, $\lambda \ne \mu$.

Any discrete triply orthogonal system consists of three (Lamé) families of *coordinate surfaces*,

$$\begin{aligned} \lambda&\mapsto \{\bar{{\mathcal {V}}} \ni (\lambda , x_2, x_3) \mapsto f_{(\lambda , x_2, x_3)}^1 \in {\mathcal {Z}} \}, \\ \lambda&\mapsto \{\bar{{\mathcal {V}}} \ni (x_1, \lambda , x_3) \mapsto f_{(x_1, \lambda ,x_3)}^2 \in {\mathcal {Z}} \}, \\ \lambda&\mapsto \{\bar{{\mathcal {V}}} \ni (x_1, x_2, \lambda ) \mapsto f_{(x_1, x_2,\lambda )}^3 \in {\mathcal {Z}} \}; \end{aligned}$$

those are discrete Legendre maps and represent discrete surfaces that intersect orthogonally along discrete curvature lines. We call those discrete lines of intersection $x_i$-*trajectories* of the system.

Due to condition (iii), any two adjacent coordinate surfaces of the same family are related by a discrete Ribaucour transformation (cf [4, 7, 30]).

In particular, for any edge of a triply orthogonal system, we obtain three distinguished spheres $(s_{ij}^\mu )_\mu$ that pairwise intersect orthogonally: two of these spheres are curvature spheres of coordinate surfaces and the third sphere is a Ribaucour sphere enveloped by the Ribaucour pair of adjacent coordinate surfaces.

Imitating the smooth notion of a cyclic system (cf [17, 18, 29]), we introduce the following notion:

#### Definition 11

A *discrete cyclic system* is a discrete triply orthogonal system such that two families of coordinate surfaces are discrete channel surfaces that intersect along their circular curvature lines.

We emphasize that the notion of a discrete triply orthogonal system as well as cyclicity of it are only invariant under Möbius transformations; hence, those notions depend on the choice of a point sphere complex.

Recall that a discrete channel surface in the sense of [23] is a discrete Legendre map that admits a constant Lie cyclide for each coordinate ribbon of one coordinate direction. Hence, the curvature spheres of the discrete channel surface along each coordinate ribbon are curvature spheres of this constant Lie cyclide.

As a consequence, a discrete channel surface has one family of circular curvature lines and one family of curvature spheres is constant along each of them. Moreover, any two adjacent circular curvature lines are curvature lines of the corresponding (constant) Lie cyclide.

Thus, since one family of (orthogonal) trajectories of a discrete cyclic system provides the circular curvature lines of the discrete channel surfaces, we conclude:

#### Corollary 12

One family of trajectories of a discrete cyclic system are circular.

Moreover, as circular curvature lines of a discrete channel surface, two adjacent trajectories are related by a discrete Ribaucour transformation that is induced by a smooth Ribaucour transformation (cf [23, Prop 2.10]). Note that this correspondence is given by an M-Lie inversion as described in Lemma 8.

Hence:

#### Corollary 13

A discrete cyclic system provides a discrete 2-dimensional congruence of circles where any two adjacent circles are cospherical.

Furthermore, for any discrete cyclic system we obtain two distinguished 2-parameter families of Dupin cyclides, namely, those that provide constant Lie cyclides for the discrete channel surfaces.

Any circle of the above congruence is then a curvature line on four Dupin cyclide patches, the Lie cyclides of the two discrete channel surfaces that intersect along this circle. Since their contact elements along the circle intersect orthogonally, the two constant curvature spheres are mutually quer-spheres of the smooth Lie cyclide patches.

### Cyclic circle congruences

In this subsection, we investigate whether a given discrete 2-dimensional circle congruence admits orthogonal surfaces and, subsequently, gives rise to a discrete cyclic system.

Thus, we consider a circle congruence on a 2-dimensional domain,

$$\begin{aligned} \Gamma = (\gamma , \gamma ^\perp ): {\mathcal {V}}\rightarrow G_{(2,1)}^{{\mathcal {P}}} \times G_{(2,1)}. \end{aligned}$$

#### Definition 14

A discrete Legendre map $f: {\mathcal {V}}\rightarrow {\mathcal {Z}}$ is said to be *orthogonal* to $\Gamma$ if, for each vertex $i \in {\mathcal {V}}$,

(i) the point sphere $f_i^\mathfrak {p}$ lies on the circle $\Gamma _i$ and
(ii) any other sphere in the contact element $f_i$ intersects the circle $\Gamma _i$ orthogonally.

Algebraically, properties (i) and (ii) amount to the following condition:

#### Lemma 15

A discrete Legendre map *f* is orthogonal to a circle congruence $\Gamma$ if and only if any element $s_i \subset f_i$ and $\mathfrak {c}_i \in \gamma _i^\perp \cap {\mathcal {L}}$ satisfy

7 $$\begin{aligned} \langle {\mathfrak {s}_i, \mathfrak {c}_i + \langle {\mathfrak {c}_i, \mathfrak {p}}\rangle \mathfrak {p}}\rangle =0 \ \ \text {for any vertex } i \in {\mathcal {V}}. \end{aligned}$$

#### Proof

Suppose that $s_i \subset f_i \cap {\mathbb {P}}({\mathcal {P}})$ is a point sphere, then equation (7) becomes $\langle {\mathfrak {s}_i, \mathfrak {c}_i}\rangle =0$. This holds if and only if the point sphere $s_i$ lies on all spheres in $\gamma _i^\perp$ and thus is a point on the circle.

For any other sphere $s_i \subset f_i$, $\langle {\mathfrak {s}_i, \mathfrak {p}}\rangle \ne 0$, the claim follows from (3). $\square$

The circle congruences that we are particularly interested in stem from discrete cyclic system:

#### Definition 16

A discrete circle congruence will be called *cyclic* if it admits a discrete family $I \ni \lambda \mapsto f^\lambda$ of orthogonal discrete Legendre maps that give rise to a discrete cyclic system.

In what follows, we will only consider *non-degenerate* circle congruences, that is, four circles of any elementary quadrilateral do not share a 1-parameter family of orthogonal spheres. By this assumption we guarantee that not all orthogonal surfaces have totally umbilic faces.

#### Theorem 17

A non-degenerate circle congruence $\Gamma =(\gamma , \gamma ^\perp ): {\mathcal {V}}\rightarrow G_{(2,1)}^{{\mathcal {P}}} \times G_{(2,1)}$ is cyclic if and only if it admits a flat connection on the trivial bundle ${\mathcal {V}}\times {\mathbb {R}}^{4,2}$ comprised of Lie inversions that map adjacent circles onto each other, that is, there exists a 2-parameter family of linear sphere complexes $a: {\mathcal {E}} \rightarrow {\mathbb {P}}({\mathbb {R}}^{4,2})$ such that the induced Lie inversions $\{\sigma _{e}\}_{e\in {\mathcal {E}}}$ satisfy

$$\begin{aligned} \sigma _{ij}(\gamma _j) = \gamma _i \ \ \text {and} \ \ \sigma _{ij} \circ \sigma _{jk} = \sigma _{il} \circ \sigma _{lk} \end{aligned}$$

for any quadrilateral (*ijkl*).

#### Remark 18

For degenerate circle congruences that admit a flat connection of this kind the result can indeed fail: consider, for example, a discrete circle congruence that consists of parallel lines such that the point spheres obtained as intersection of the lines with a fixed orthogonal plane do not form a 2-dimensional circular net. Then, this circle congruence does not give rise to a discrete cyclic system, because there are no orthogonal surfaces that are discrete Legendre maps. However, the reflections in the bisecting planes of two adjacent parallel lines provide a flat connection in the sense of Theorem 17.

#### Proof

Suppose that the circle congruence $\Gamma : {\mathcal {V}}\rightarrow G_{(2,1)}^{{\mathcal {P}}} \times G_{(2,1)}$ is cyclic. Then, by definition, there exists an associated discrete cyclic system $f=(f^1,f^2,f^3)$ on ${\mathcal {V}}\times I$, where $f^1$ and $f^2$ denote the two coordinate surface families of discrete channel surfaces.

These discrete channel surfaces induce a canonical M-Lie inversion between two adjacent circles $\Gamma _i$ and $\Gamma _j$ of $\Gamma$: namely, the M-Lie inversion that interchanges the curvature circles of the constant Lie cyclide provided by one of the discrete channel surfaces, $f^1$ or $f^2$. We denote these M-Lie inversions by $\sigma _{ij}$ and obtain: $\sigma _{ij}(\gamma _j)=\gamma _i$.

Moreover, by construction, these M-Lie inversions map adjacent contact elements (frames) $f_i$ and $f_j$ of the discrete cyclic system onto each other. Since the orthogonal surfaces $f^3$ are discrete Legendre maps, the connection provided by $\sigma$ is flat on $\gamma$. Furthermore, since, for any $i \in {\mathcal {V}}$ and $m \in I$, we have $f_{i,m}^\mu \cap \gamma _i^\perp =: s_i^\mu \in {\mathbb {P}}({\mathcal {L}})$, $\mu =1,2$, it follows that

$$\begin{aligned} (\sigma _{ij} \circ \sigma _{jk} \circ \sigma _{kl} \circ \sigma _{li})(s_i^\mu ) = s_i^\mu . \end{aligned}$$

Together with the fact that the M-Lie inversions $\sigma$ preserve the point sphere complex $\mathfrak {p}$, we conclude that the induced connection is also flat on $\gamma ^\perp$.

Conversely, assume that a circle congruence $\Gamma$ admits a flat connection provided by Lie inversions $\sigma _{ij}$ that map adjacent circles $\gamma _i$ and $\gamma _j$ onto each other, that is, any point sphere in $\gamma _i$ is mapped to a point sphere in $\gamma _j$. Thus the Lie inversion $\sigma _{ij}$ preserves the point sphere complex $\mathfrak {p}$ and is an M-Lie inversion.

To prove that $\Gamma$ is indeed cyclic, we will construct a discrete family $I \ni \lambda \mapsto f^{3,\lambda }$ of orthogonal surfaces that is part of a discrete cyclic system. To do so, we fix point spheres $I \ni \lambda \mapsto p^\lambda _0$ along an initial circle $\Gamma _{i_0}$ of the circle congruence. Furthermore, according to Fact 4, we choose contact elements $\lambda \mapsto f^{3,\lambda }_{i_0}$ that consist of spheres orthogonal to $\Gamma _{i_0}$ such that any two adjacent contact elements share a common sphere (cf Fig. 1).

Then transport of these contact elements $f_{i_0}^{3,\lambda }$ along edges $ij\in {\mathcal {E}}$ by the M-Lie inversions $\sigma _{ij}$ consecutively defines a discrete family of orthogonal surfaces $\lambda \mapsto f^{3,\lambda }$ by

$$\begin{aligned} f_j^{3,\lambda } := \sigma _{ji}(f_i^{3,\lambda }). \end{aligned}$$

Due to the flatness of the connection, this construction is well defined. Moreover, since any M-Lie inversion $\sigma _{ij}$ fixes a (curvature) sphere $s_{ij}^\lambda \subset f_i^{3,\lambda } \cap f_j^{3,\lambda }$ and the circle congruence is non-degenerate, we indeed obtain discrete Legendre maps $f^{3,\lambda }$.

To complete the proof, it remains to construct two families $\lambda _1 \mapsto f^{1,\lambda _1}$ and $\lambda _2 \mapsto f^{2,\lambda _2}$ of discrete channel surfaces that supplement the family $f^{3,\lambda }$ of orthogonal surfaces. To equip the point sphere maps with suitable contact elements, we choose two spheres $\mathfrak {r}_{i_0}^1, \mathfrak {r}_{i_0}^2 \in \gamma _{i_0}^\perp \cap {\mathcal {L}}$ that intersect each other orthogonally and define contact elements along the circle $\Gamma _{i_0}$ by

$$\begin{aligned} f^{\mu , i_0}_{\lambda } : = \text {span}\{ {\mathfrak {r}_{i_0}^\mu , p_{0}^\lambda }\} \ \ \text {for } \ \mu \in \{ 1,2\}. \end{aligned}$$

Then transport of these two contact elements by means of the M-Lie inversions $\sigma$ along edges provides two families of discrete channel surfaces, with the circles of the congruence as curvature lines.

Thus, in summary, the constructed map $f:=(f^1, f^2, f^3)$ provides a discrete cyclic system with underlying circle congruence $\Gamma$. $\square$

Hence, to any discrete cyclic system we associate a unique 2-parameter family of M-Lie inversions $\sigma _{ij}$, defined on edges of the associated circle congruence, which simultaneously map adjacent point spheres of all orthogonal surfaces onto each other. Note that these M-Lie inversions are symmetric, that is, $\sigma _{ij}=\sigma _{ji}$.

These M-Lie inversions induce a Ribaucour correspondence between any two adjacent circles, which are therefore cospherical. Furthermore, they reveal a property well-known from smooth cyclic systems:

#### Corollary 19

The cross-ratio of the point spheres of any four orthogonal surfaces of a discrete cyclic system is constant along the orthogonal surfaces.

Moreover, by using Fact 4, we observe another relation between any two orthogonal surfaces of a discrete cyclic system (see also Fig. 1).

#### Corollary 20

Up to a possible change of orientation, any two orthogonal surfaces of a discrete cyclic system are related by a discrete Ribaucour transformation.

**Fig. 2:**
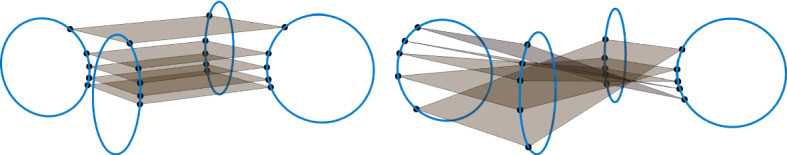
Contrary to the smooth case, the family of orthogonal surfaces to a discrete cyclic circle congruence is not necessarily unique

However we note that, in contrast to the smooth theory, the reconstruction of a discrete cyclic system from a cyclic circle congruence is not unique. There are several ambiguities: firstly, a discrete cyclic circle congruence can admit various flat connections in the sense of Theorem 17. This non-uniqueness of the connection stems from the generic existence of two Ribaucour correspondences between two cospherical circles (cf Lemma 8). An example of different flat connections associated with a face of a discrete cyclic circle congruence is illustrated in Fig. 2.

Hence a discrete circle congruence does generically not have a unique family of orthogonal surfaces, even after an initial circle is equipped with a point sampling.

Secondly, once a discrete family of orthogonal surfaces is established, we are left with a 1-parameter choice for the other two families of coordinate surfaces, namely the discrete channel surfaces. Although the circular curvature lines are already fixed, we have the choice of a pair of orthogonally intersecting curvature spheres for the discrete channel surfaces at an initial circle (see the choice of the spheres $r_{i_0}^1$ and $r_{i_0}^2$ in the proof of Theorem 17).

Recall [20, 24, 29] that, in the smooth case, the existence of three orthogonal surfaces of a circle congruence already implies that the congruence is cyclic. We obtain an analogous statement in the discrete case.

We say that three orthogonal discrete Legendre maps are *generic* if, for any edge of the circle congruence, the three curvature spheres span a (2, 1)-plane of ${\mathbb {R}}^{4,2}$.

#### Theorem 21

A discrete circle congruence that admits three generic orthogonal discrete Legendre maps is cyclic.

#### Proof

Let $\Gamma :{\mathcal {V}}\rightarrow G_{(2,1)}^{{\mathcal {P}}} \times G_{(2,1)}$ be a circle congruence that admits at least three generic orthogonal discrete Legendre maps $g^\mu$, $\mu \in \{1,2,3 \}$. To prove that $\Gamma$ is cyclic, we will construct a flat connection as described in Theorem 17.

Firstly, consider two adjacent circles $\Gamma _i$ and $\Gamma _j$ of the congruence $\Gamma$ and denote by

$$\begin{aligned} \mathfrak {s}_i, \mathfrak {t}_i \in \gamma _i^\perp \cap {\mathcal {L}}\end{aligned}$$

two spheres that intersect in the circle $\Gamma _i$, and similarly for $\Gamma _j$. Since the curvature spheres $s_{ij}^\mu$ of the orthogonal surfaces that belong to the edge (*ij*) intersect all spheres in $\gamma _i^\perp$ and $\gamma _j^\perp$ orthogonally we obtain

8 $$\begin{aligned} s_{ij}^\mu \subset \text {span}\{ {\mathfrak {s}_1 + \langle {\mathfrak {s}_1, \mathfrak {p}}\rangle \mathfrak {p}, \ \mathfrak {s}_2 + \langle {\mathfrak {s}_2, \mathfrak {p}}\rangle \mathfrak {p}, \ \mathfrak {t}_1 + \langle {\mathfrak {t}_1, \mathfrak {p}}\rangle \mathfrak {p}, \ \mathfrak {t}_2 + \langle {\mathfrak {t}_2, \mathfrak {p}}\rangle \mathfrak {p}}\} ^\perp . \end{aligned}$$

Hence we deduce that this span is at most 3-dimensional, which implies that the space

$$\begin{aligned} \text {span}\{ {\mathfrak {s}_1, \mathfrak {s}_2, \mathfrak {t}_1, \mathfrak {t}_2, \mathfrak {p}}\} \subset {\mathbb {R}}^{4,2} \end{aligned}$$

is at most 4-dimensional. Therefore, due to Fact 5, the two circles $\Gamma _1$ and $\Gamma _2$ lie on a common sphere. This sphere is unique up to orientation.

As a consequence, using Corollary 9, there exists a unique Ribaucour correspondence between any two adjacent circles that extends the correspondence induced by the point sphere maps of the orthogonal surfaces $\{g^\mu \}_\mu$. By Lemma 8, this correspondence is described by an M-Lie inversion $\sigma _{ij}$.

Since $\{g^\mu \}_\mu$ are discrete Legendre maps, these M-Lie inversions provide a flat connection for $\Gamma$. Consequently, the circle congruence $\Gamma$ is indeed cyclic. $\square$

To illustrate the situation we investigate a standard example for cyclic circle congruences, namely, those that stem from a parallel family of surfaces in a space form. To start with, we discuss discrete parallel surfaces in a flat ambient space form as described in [3]:

#### Example 22

Fix a point sphere complex $\mathfrak {p}$ and a flat space form by choosing a lightlike space form vector $q \in {\mathbb {P}}({\mathcal {L}})$, $\langle {\mathfrak {p}, \mathfrak {q}}\rangle =0$. Let $f:{\mathcal {V}}\rightarrow {\mathcal {Z}}$ be a discrete Legendre map with space form projection

$$\begin{aligned} (\mathfrak {f}^\mathfrak {p}, \mathfrak {t}): {\mathcal {V}}\rightarrow {\mathcal {Q}}\times {\mathcal {H}}, \end{aligned}$$

where $\mathfrak {f}^\mathfrak {p}$ and $\mathfrak {t}$ denote the point sphere map and tangent plane congruence in *f*, respectively. Moreover, we denote the family of parallel discrete Legendre maps of *f* by $I \ni \lambda \rightarrow (\mathfrak {f}^{\mathfrak {p},\lambda }, \mathfrak {t^\lambda })$.

At each vertex, the point spheres of the parallel family lie on the line orthogonal to the tangent plane. Using Fact 6, this line is described by

$$\begin{aligned} \mathfrak {f}_i^{\mathfrak {p}, \lambda } \subset \text {span}\{ {\mathfrak {f}_i^\mathfrak {p}, \mathfrak {q}, \mathfrak {t}_i + \langle {\mathfrak {t}_i, \mathfrak {p}}\rangle \mathfrak {p}}\} =: \gamma _i. \end{aligned}$$

Moreover, note that the point spheres $\mathfrak {f}_i^{\mathfrak {p}, \lambda }$ and $\mathfrak {f}_j^{\mathfrak {p}, \lambda }$ of adjacent vertices, as well as adjacent tangent planes, are related by the reflection in the corresponding bisecting hyperplane. Hence the sphere complexes

$$\begin{aligned} a:{\mathcal {E}} \rightarrow {\mathbb {P}}({\mathcal {P}}), \ \mathfrak {a}_{ij} : = \mathfrak {t}_i - \mathfrak {t}_j \end{aligned}$$

provide a flat connection for the discrete line congruence $\Gamma =(\gamma , \gamma ^\perp )$, which is therefore cyclic.

The two further coordinate surface families of a discrete cyclic system associated with this line congruence consist of developable discrete channel surfaces. They are determined by two suitably prescribed curvature spheres at one initial vertex of the discrete line congruence, that is, by the choice of two orthogonal planes that intersect in the line of the congruence at this initial vertex.

To conclude this section, we emphasize that our analysis also leads to a notion of *semi-discrete cyclic systems*, where the orthogonal surfaces are discrete Legendre maps and the other two coordinate surface families consist of semi-discrete channel surfaces — as has already become evident from Example 22.

In particular, any discrete cyclic circle congruence gives rise to semi-discrete cyclic systems: once a flat connection in the sense of Theorem 17 is established, we choose a (smooth) 1-parameter family of orthogonal contact elements along an initial circle, as well as two suitable curvature spheres for the semi-discrete channel surfaces that orthogonally intersect in this initial circle. The transport of this initial principal frame by means of the M-Lie inversions of the connection then gives rise to the sought-after semi-discrete cyclic system.

## Discrete cyclic systems with special orthogonal surfaces

Historically, smooth cyclic systems were closely related to the Ribaucour transformation of surfaces, as described by Ribaucour [29, 32]: the circle congruence formed by the circles that orthogonally intersect the surfaces of a Ribaucour pair in corresponding points is cyclic; furthermore, any two orthogonal surfaces of this cyclic circle congruence form a Ribaucour pair.

By imposing special geometric properties on a Ribaucour pair of surfaces, this construction gives rise to particular cyclic systems. Among them are cyclic systems with orthogonal surfaces that are Guichard surfaces [1, 9] and, in particular, parallel families of flat fronts in hyperbolic space [11]; and cyclidic systems, where all coordinate surfaces are Dupin cyclides [18, 33].

We report on a similar construction in the discrete framework: the orthogonal circle congruence of a discrete Ribaucour pair is cyclic. Subsequently, we investigate circle congruences constructed from discrete Ribaucour pairs with (at least) one totally umbilic discrete envelope. In this way, we obtain discrete cyclic circle congruences with discrete flat fronts in hyperbolic space as orthogonal surfaces, as well as discrete cyclic systems where all coordinate surfaces are discrete Dupin cyclides. Cyclic systems with a 1-parameter family of orthogonal Guichard surfaces will be reported on in a forthcoming paper.

In this section we only consider *non-degenerate* discrete Ribaucour sphere congruences $r:{\mathcal {V}} \rightarrow {\mathbb {P}}({\mathcal {L}})$ (cf [4]), that is, discrete conjugate nets (nets with planar faces) in ${\mathbb {P}}({\mathcal {L}})$, where any homogeneous coordinate vectors of each face span a (2, 1)- or a (1, 2)-plane. In the former case, where every span has Minkowski signature, each face of the sphere congruence models a Dupin cyclide, which we will refer to as the associated *R-Dupin cyclide*.

The notion of a Ribaucour sphere congruence is clearly a Lie sphere geometric notion. However, once we start to construct discrete cyclic circle congruences, we again fix a Möbius subgeometry of Lie sphere geometry, modelled on a point sphere complex $\mathfrak {p}$. We say that a discrete Ribaucour sphere congruence is *non-degenerate in this Möbius subgeometry* if it additionally satisfies $\langle {\mathfrak {r}_i, \mathfrak {p}}\rangle \ne 0$, that is, if it does not contain any point spheres.

**Fig. 3:**
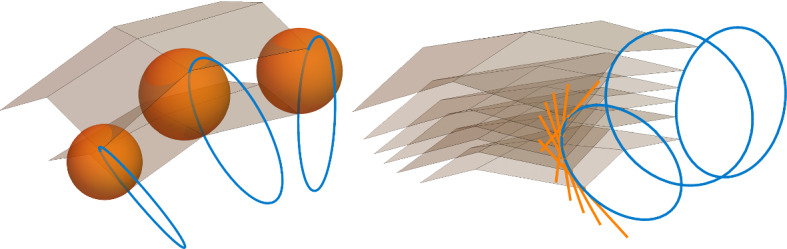
Left: A Ribaucour pair of discrete Legendre maps with a part of its associated circle congruence (blue), consisting of circles that intersect the spheres of the Ribaucour congruence (orange) orthogonally in the point spheres of the envelopes. Right: Discrete Legendre maps that are orthogonal to the circle congruence associated with the Ribaucour pair (colour figure online)

### Discrete cyclic circle congruences associated with discrete Ribaucour pairs

We begin by formulating useful general observations about Ribaucour sphere congruences: first we provide a characterization in terms of the existence of certain flat connections, then show that a flat connection can be chosen to be comprised of M-Lie inversions once a Möbius subgeometry is fixed. Given a pair of envelopes of the Ribaucour sphere congruence, this connection then also yields a flat connection for the orthogonal circle congruence that we are interested in, showing that it is cyclic (cf Thm 17).

Recall [4, 30] that any non-degenerate discrete Ribaucour sphere congruence admits a 2-parameter family of envelopes, which are uniquely determined by the choice of one appropriate contact element at one initial Ribaucour sphere.

Conversely, two discrete Legendre maps form a discrete Ribaucour pair if they envelop a common sphere congruence; this sphere congruence is then a discrete Ribaucour sphere congruence.

Taking the first point of view and focusing on the sphere congruence, we obtain the following characterization of discrete Ribaucour sphere congruences:

#### Proposition 23

A discrete sphere congruence $r: {\mathcal {V}} \rightarrow {\mathbb {P}}({\mathcal {L}})$ with $\langle {\mathfrak {r}_i, \mathfrak {r}_j}\rangle \ne 0$ for $(ij) \in {\mathcal {E}}$, is a discrete Ribaucour sphere congruence if and only if it admits a flat connection on the trivial bundle ${\mathcal {V}} \times {\mathbb {R}}^{4,2}$ comprised of Lie inversions that map adjacent Ribaucour spheres onto each other.

#### Proof

Let $r: {\mathcal {V}} \rightarrow {\mathbb {P}}({\mathcal {L}})$ be a discrete Ribaucour sphere congruence and fix $n \in {\mathbb {P}}({\mathbb {R}}^{4,2})$ such that $\langle {\mathfrak {n}, \mathfrak {r}_i}\rangle \ne 0$ for any $i \in {\mathcal {V}}$. We consider the discrete map

9 $$\begin{aligned} a:{\mathcal {E}} \rightarrow {\mathbb {P}}({\mathbb {R}}^{4,2}), (ij)\mapsto a_{ij} := \text {span}\{ {\mathfrak {a}_{ij}}\},\;\; \text {where}\;\;\mathfrak {a}_{ij} := \langle {\mathfrak {n}, \mathfrak {r}_i}\rangle \mathfrak {r}_j - \langle {\mathfrak {n}, \mathfrak {r}_j}\rangle \mathfrak {r}_i \end{aligned}$$

and denote the Lie inversions with respect to the linear sphere complex $a_{ij}$ by $\sigma _{ij}$. By construction, those Lie inversions exchange adjacent spheres of the congruence and satisfy, for any face (*ijkl*),

$$\begin{aligned} (\sigma _{ij}\circ \sigma _{jk}\circ \sigma _{kl}\circ \sigma _{li}) (\mathfrak {r}_i) = \mathfrak {r}_i. \end{aligned}$$

To prove that the Lie inversions $\sigma$ indeed yield a flat connection on ${\mathcal {V}} \times {\mathbb {R}}^{4,2}$, we will investigate the interplay between the 2-parameter family of envelopes of *r* and these Lie inversions. Firstly, note that $\sigma _{ij}$ preserves all spheres that are in oriented contact with $r_i$ and $r_j$; hence, adjacent contact elements of the envelopes are exchanged (see also [30, §3]). In particular, we obtain that $(\sigma _{ij}\circ \sigma _{jk}\circ \sigma _{kl}\circ \sigma _{li}) (f_i) = f_i$ for the contact elements of any envelope *f*.

Moreover, since $\langle {\mathfrak {a}_{ij}, \mathfrak {n}}\rangle =0$, the Lie inversions preserve the linear sphere complex ${\mathbb {P}}({\mathcal {L}} \cap n^\perp )$. Since each contact element of an envelope contains exactly one sphere in ${\mathbb {P}}({\mathcal {L}} \cap n^\perp )$ and any discrete Ribaucour sphere congruence admits a 2-parameter family of discrete envelopes, we conclude that, for any $\mathfrak {v} \in r_i^\perp$,

$$\begin{aligned} (\sigma _{ij}\circ \sigma _{jk}\circ \sigma _{kl}\circ \sigma _{li}) (\mathfrak {v})=\mathfrak {v}. \end{aligned}$$

Thus, the Lie inversions $\sigma _{ij}$ provide a flat connection for the Ribaucour sphere congruence.

Conversely, assume that $r:{\mathcal {V}} \rightarrow {\mathbb {P}}({\mathcal {L}})$, $\langle {\mathfrak {r}_i, \mathfrak {r}_j}\rangle \ne 0$, is a discrete sphere congruence that admits a flat connection comprised of Lie inversions, as above. Furthermore, let $s_0 \in {\mathbb {P}}({\mathcal {L}})$ be a sphere in oriented contact to an initial sphere $r_0$ of the Ribaucour sphere congruence, that is, $s_0 \perp r_0$. Then propagation of the contact element $\text {span}\{ {s_0, r_0}\}$ by means of the flat connection provides a well-defined discrete Legendre map that envelopes *r*. Due to the possible choices for $s_0$, we obtain a 2-parameter family of discrete envelopes and *r* is indeed a discrete Ribaucour sphere congruence. $\square$

We remark that the flat connection of Prop 23 is not unique. However, if we fix a Möbius subgeometry by choosing a point sphere complex $\mathfrak {p}$ such that the Ribaucour sphere congruence is non-degenerate in this Möbius subgeometry, we may fix homogeneous coordinates $\mathfrak {r}:{\mathcal {V}}\rightarrow {\mathcal {L}}$ so that $\langle {\mathfrak {r},\mathfrak {p}}\rangle \equiv 1$ and consider the discrete map

10 $$\begin{aligned} a:{\mathcal {E}} \rightarrow {\mathbb {P}}({\mathbb {R}}^{4,2}), (ij)\mapsto a_{ij} := \text {span}\{ {\mathfrak {a}_{ij}}\},\;\; \text {where}\;\; \mathfrak {a}_{ij} := \mathfrak {r}_j - \mathfrak {r}_i. \end{aligned}$$

Then the associated Lie inversions $\sigma _{ij}$ preserve the point sphere complex and are therefore M-Lie inversions (cf [30, §3.1]). Hence:

#### Corollary 24

A discrete sphere congruence $r: {\mathcal {V}} \rightarrow {\mathbb {P}}({\mathcal {L}})\setminus {\mathbb {P}}({\mathcal {P}})$ with $\langle {\mathfrak {r}_i, \mathfrak {r}_j}\rangle \ne 0$ for $(ij) \in {\mathcal {E}}$, is a discrete Ribaucour sphere congruence if and only if it admits a (unique) flat connection on the trivial bundle ${\mathcal {V}} \times {\mathbb {R}}^{4,2}$ compound by M-Lie inversions that map adjacent Ribaucour spheres onto each other.

Returning to our principal aim, the construction of a cyclic circle congruence associated with a discrete Ribaucour pair, we fix two discrete Legendre maps $(f^+, f^-):{\mathcal {V}} \rightarrow {\mathcal {Z}}\times {\mathcal {Z}}$ that envelop the non-degenerate discrete Ribaucour sphere congruence $r:{\mathcal {V}} \rightarrow {\mathbb {P}}({\mathcal {L}})\setminus {\mathbb {P}}({\mathcal {P}})$.

Then the flat connection of M-Lie inversions associated with *r* (see Cor 24) will also provide a flat connection for the circle congruence: we consider the circle congruence $\Gamma :{\mathcal {V}} \rightarrow G_{(2,1)}^{{\mathcal {P}}} \times G_{(2,1)}$ consisting of circles $\Gamma _i$ that intersect the spheres $r_i$ in the point spheres $p^\pm _i \subset f^\pm _i$ of the envelopes orthogonally (for an illustration see Fig. 3). By Fact 6, this circle congruence is described by the (2, 1)-planes

11 $$\begin{aligned} \gamma _i:=\text {span}\{ {\mathfrak {p}_i^+, \ \mathfrak {p}_i^-, \ \mathfrak {r}_i+\mathfrak {p}}\} . \end{aligned}$$

Since the M-Lie inversions $\sigma$ described by (10) satisfy $\sigma _{ij}(\mathfrak {r}_j + \mathfrak {p})=\mathfrak {r}_i+ \mathfrak {p}$, they also map adjacent circles of $\Gamma$ onto each other, that is, $\sigma _{ij}(\gamma _j)=\gamma _i$.

Thus, by the above, these M-Lie inversions $\sigma$ yield a flat connection for the circle congruence $\Gamma$ and, by Theorem 17, we conclude:

#### Theorem 25

Let $p^\pm \subset f^\pm$ be the point sphere maps of two envelopes $f^\pm$ of a discrete Ribaucour sphere congruence $r:{\mathcal {V}}\rightarrow {\mathbb {P}}({\mathcal {L}})$. Then the circles that orthogonally intersect the spheres *r* in the point sphere maps $p^\pm$ form a cyclic circle congruence with $f^\pm$ as orthogonal surfaces. This circle congruence is given by

$$\begin{aligned} \begin{aligned} \Gamma =(\gamma , \gamma ^\perp ): {\mathcal {V}}&\rightarrow G_{(2,1)}^{{\mathcal {P}}} \times G_{(2,1)}, \\ i&\mapsto \text {span}\{ {\mathfrak {p}_i^+, \mathfrak {p}_i^-, \mathfrak {r}_i+\mathfrak {p}}\} \times \text {span}\{ {\mathfrak {p}_i^+, \mathfrak {p}_i^-, \mathfrak {r}_i+\mathfrak {p}}\} ^\perp , \end{aligned} \end{aligned}$$

where $\mathfrak {r}_i \in r_i$ such that $\langle {\mathfrak {r}_i, \mathfrak {p}}\rangle =1$; it will be referred to as *associated* to the discrete Ribaucour pair.

Firstly, we will exploit this construction to extend Example 22 and discuss discrete (normal) line congruences in space forms obtained from parallel discrete surfaces.

#### Example 26

*(Discrete cyclic circle congruences associated to parallel surfaces in space forms)* Let $\mathfrak {p} \in {\mathbb {R}}^{4,2}$, $\langle {\mathfrak {p}, \mathfrak {p}}\rangle =-1$, be a fixed point sphere complex and $\mathfrak {q} \in {\mathbb {R}}^{4,2}\setminus \{ 0 \}$, $\langle {\mathfrak {p}, \mathfrak {q}}\rangle =0$, a space form vector satisfying $\langle {\mathfrak {q},\mathfrak {q}}\rangle =\pm 1$. As before, we denote the space form projection of a discrete Legendre map *f* by

$$\begin{aligned} (\mathfrak {f}^\mathfrak {p}, \mathfrak {t}): {\mathcal {V}}\rightarrow {\mathcal {Q}}\times {\mathcal {H}}, \end{aligned}$$

with the point sphere map $\mathfrak {f}^\mathfrak {p}$ and tangent plane congruence $\mathfrak {t}$ of *f*, respectively. Furthermore, consider its discrete Ribaucour transform ${\hat{f}}:=\sigma _q(f)$ obtained by the Lie inversion in the linear sphere complex ${\mathbb {P}}({\mathcal {L}}\cap \{ \mathfrak {q}\}^\perp )$.

By Theorem 25, the discrete cyclic circle congruence associated with the Ribaucour pair $(f, {\hat{f}})$ is given by

$$\begin{aligned} \gamma _i : = \text {span}\{ {\mathfrak {f}_i^p, \hat{\mathfrak {f}}_i^p, \mathfrak {t}_i+\langle {\mathfrak {t}_i, \mathfrak {p}}\rangle \mathfrak {p}}\} = \text {span}\{ {\mathfrak {f}_i^p, \mathfrak {q}, \mathfrak {t}_i-\mathfrak {p}}\} , \end{aligned}$$

which yields a discrete normal line congruence in the distinguished space form, which admits the family of parallel surfaces as its orthogonal surfaces.

In the case of a hyperbolic ambient quadric of constant curvature, that is, $\langle {\mathfrak {q}, \mathfrak {q}}\rangle > 0$, the circles of the constructed congruence $\Gamma$ orthogonally intersect the spheres $\mathfrak {l}^\pm = \mathfrak {p} \pm \mathfrak {q}$ that coincide up to orientation. Those spheres $\mathfrak {l}^\pm$ represent the infinity boundary of the hyperbolic quadric of constant curvature, which consists of two hyperbolic space forms. Moreover, the two orthogonal surfaces whose point spheres lie on $l^\pm$ provide the two discrete hyperbolic Gauss maps of *f* and its parallel surfaces.

Thus, by Corollary 20, we learn that the two discrete hyperbolic Gauss maps of a discrete Legendre map are (up to orientation) related by a discrete Ribaucour transformation. This fact suggests another construction for discrete cyclic circle congruences associated with parallel surfaces in hyperbolic space, namely, from its two discrete hyperbolic Gauss maps.

We say that a discrete Legendre map is *totally umbilic* if all curvature spheres coincide and, for any choice of point sphere complex, its point sphere map is a circular net.

Using this definition, we have proven:

#### Corollary 27

A discrete Ribaucour pair of two totally umbilic discrete Legendre maps whose point spheres lie on the same sphere gives rise to a discrete normal line congruence in an appropriate hyperbolic space form. Any of its orthogonal surfaces in this hyperbolic space form are parallel surfaces and have the discrete Ribaucour pair as their common discrete hyperbolic Gauss maps.

Clearly, the geometry of the chosen discrete hyperbolic Gauss maps affects the properties of the associated orthogonal surfaces. For example, in the next subsection, we shall see that starting with hyperbolic Gauss maps that form a Darboux pair will lead to discrete flat fronts in hyperbolic space.

### Discrete flat fronts as orthogonal surfaces of cyclic circle congruences

In the smooth, as well as in the discrete setup, there are various ways to construct intrinsically flat surfaces (or fronts) in hyperbolic space, that is, surfaces with constant extrinsic Gaussian curvature 1.

From a Lie sphere geometric perspective, smooth and discrete flat fronts in hyperbolic space are obtained as projections of $\Omega$-surfaces spanned by two isothermic sphere congruences each lying in a fixed parabolic linear sphere complex (see [11, 12]). Alternatively, as discussed in [21, 25, 31], smooth and discrete flat fronts can be produced from holomorphic data by means of a Weierstrass type representation.

In [11], it was shown that smooth flat fronts also arise as orthogonal surfaces of special cyclic systems associated with Darboux pairs of totally umbilic surfaces, namely, of their two hyperbolic Gauss maps. The aim of this subsection is to demonstrate a similar construction within the framework developed here, which also leads to parallel families of discrete flat fronts in hyperbolic space.

In order to make contact with our present setting, we briefly recall the Lie geometric approach to discrete flat fronts in hyperbolic space as established in [12, Expl 4.3]: a discrete $\Omega$-surface is a Legendre map $f=s^+\oplus s^-$ that is spanned by a suitable pair of isothermic sphere congruences, and its hyperbolic space form projection $(\mathfrak {f}^\mathfrak {p},\mathfrak {t}): {\mathcal {V}}\rightarrow {\mathcal {Q}}\times {\mathcal {H}}$ with respect to the point sphere complex $\mathfrak {p}$ and space form vector $\mathfrak {q}$ is a flat front if and only if the enveloped isothermic sphere congruences take values in two fixed parabolic linear sphere complexes $l^\pm =\text {span}\{ {\mathfrak {q}\mp \mathfrak {p}}\}$, that is, $s^\pm \perp l^\pm$.

Fixing homogeneous coordinate vectors $\mathfrak {l}^\pm =\mathfrak {q}\mp \mathfrak {p}$, Königs dual lifts of the two sphere congruences $s^\pm$ are determined by

$$\begin{aligned} \mathfrak {s}^\pm = \pm ( \langle {\mathfrak {l}^\pm ,\mathfrak {t}}\rangle \mathfrak {f}^\mathfrak {p} - \langle {\mathfrak {l}^\pm ,\mathfrak {f}^\mathfrak {p}}\rangle \mathfrak {t} ) = \mathfrak {f}^\mathfrak {p} \pm \mathfrak {t}; \end{aligned}$$

that is, they are edge-parallel and opposite diagonals on each face are parallel:

$$\begin{aligned} \mathfrak {s}_i^+-\mathfrak {s}_j^+ \parallel \mathfrak {s}_i^--\mathfrak {s}_j^-, \mathfrak {s}_i^+-\mathfrak {s}_k^+ \parallel \mathfrak {s}_j^--\mathfrak {s}_l^- \;\; \text {and}\;\; \mathfrak {s}_j^+-\mathfrak {s}_l^+ \parallel \mathfrak {s}_k^--\mathfrak {s}_i^-. \end{aligned}$$

The former condition is equivalently expressed by the vanishing of the mixed area on faces,

$$\begin{aligned} 0 = A(\mathfrak {s}^+,\mathfrak {s}^-), \;\; \text{while}\;\; A(\mathfrak {s}^+,\mathfrak {s}^-) = A(\mathfrak {f}^\mathfrak {p},\mathfrak {f}^\mathfrak {p}) - A(\mathfrak {t},\mathfrak {t}), \end{aligned}$$

showing that the mixed area Gauss curvature $K:=\frac{A(\mathfrak {t},\mathfrak {t})}{A(\mathfrak {f}^\mathfrak {p},\mathfrak {f}^\mathfrak {p})}$ of $(\mathfrak {f}^\mathfrak {p},\mathfrak {t})$ satisfies $K\equiv 1$ if and only if $\mathfrak {s}^\pm$ are Königs dual.

The key point in the construction of discrete cyclic circle congruences that admit a parallel family of discrete flat fronts is the interplay between the distinguished isothermic sphere congruences of the discrete flat fronts and their hyperbolic Gauss maps as totally umbilic envelopes of them.

The proposed construction will rely on the following general observations on discrete Ribaucour sphere congruences with a totally umbilic (discrete) envelope:

#### Lemma 28

(cf [30]) Let *r* be a discrete Ribaucour sphere congruence that admits a totally umbilic envelope, then *r* is a (2, 1)-congruence and, on any face, the contact elements of the totally umbilic envelope coincide with contact elements of the corresponding R-Dupin cyclide along one circular curvature line of it.

In this situation, the cross-ratios of the discrete Ribaucour congruence are transferred to the point sphere map of the totally umbilic envelope and vice versa. This follows from a simple fact about smooth Dupin cyclides:

#### Lemma 29

The cross-ratio of four point spheres lying on a curvature line of a Dupin cyclide coincides with the cross-ratio of the four (non-constant) curvature spheres of the Dupin cyclide that are in oriented contact with those point spheres.

#### Proof

Let $\Delta =\delta _1 \oplus _\perp \delta _2 \in G_{(2,1)} \times G_{(2,1)}$ represent a Dupin cyclide; further let $\mathfrak {s}_1\in \delta _1\cap {\mathcal {L}}$ and $\mathfrak {s}_{2j}\in \delta _2\cap {\mathcal {L}}$ ($j=1,\dots ,4$) denote one, respectively four, curvature spheres of different families, that is, $f_j=s_1\oplus s_{2j}$ ($j=1,\dots ,4$) yield four contact elements of the cyclide. The point spheres of the corresponding contact elements are then given by

$$\begin{aligned} \mathfrak {p}_j = \langle {\mathfrak {p},\mathfrak {s}_1}\rangle \mathfrak {s}_{2j} - \langle {\mathfrak {p},\mathfrak {s}_{2j}}\rangle \mathfrak {s}_1. \end{aligned}$$

As $\langle {\mathfrak {p}_i,\mathfrak {p}_j}\rangle =\langle {\mathfrak {s}_{2i},\mathfrak {s}_{2j}}\rangle$ we conclude that $cr(p_1,p_2,p_3,p_4)=cr(s_{21},s_{22},s_{23},s_{24})$. $\square$

As a consequence of Lemmas 28 and 29 we then obtain:

#### Corollary 30

The face cross-ratios of a discrete Ribaucour sphere congruence and of a totally umbilic envelope coincide. In particular, a totally umbilic envelope of a discrete Ribaucour sphere congruence is isothermic if and only if the sphere congruence is.

**Fig. 4:**
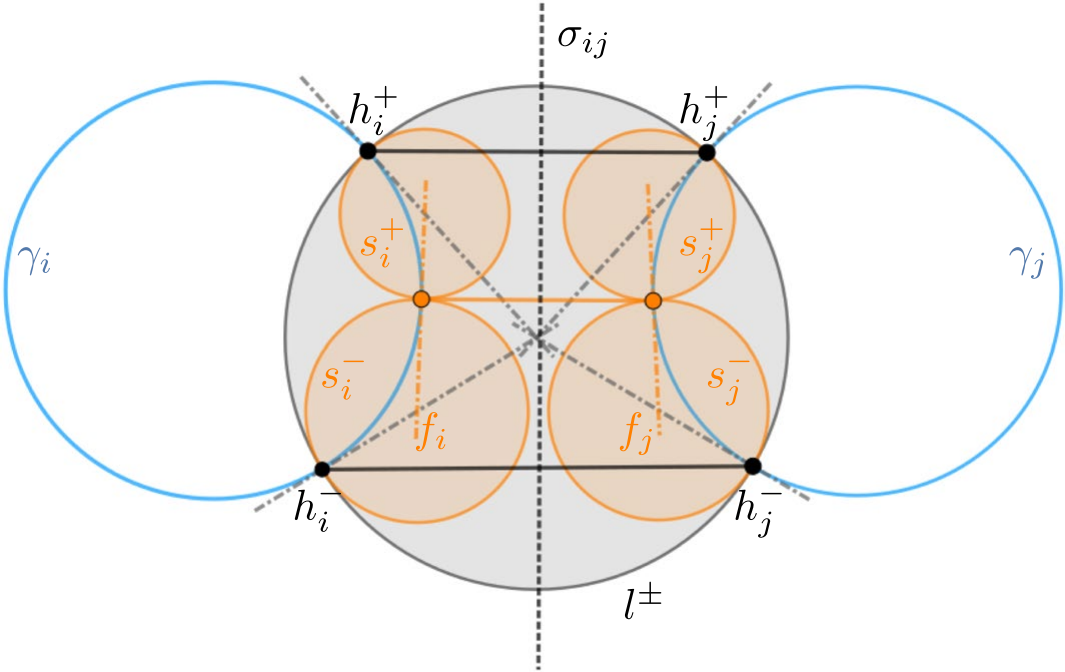
Construction of a discrete flat front $f=\text {span}\{ {s^+, s^-}\}$ from the discrete cyclic circle congruence $\Gamma$ associated with a discrete Darboux pair of totally umbilic point sphere maps $h^\pm$ with values in the fixed sphere $l^\pm$. Those become the point sphere maps of the two hyperbolic Gauss maps of the discrete flat front

In view of the fact that a discrete flat front is spanned by a pair of isothermic sphere congruences, and those are discrete Ribaucour sphere congruences that each have one of the discrete hyperbolic Gauss maps as totally umbilic second envelopes, we state the following theorem (see also Fig. 4):

#### Theorem 31

The hyperbolic Gauss maps $h^\pm :{\mathcal {V}}\rightarrow {\mathbb {P}}({\mathcal {P}})$ of a discrete flat front in hyperbolic space form a (totally umbilic) Darboux pair.

Conversely, any orthogonal net of the cyclic circle congruence associated with a Darboux pair $(h^+,h^-)$ with values in a 2-sphere projects to a flat front in the hyperbolic space bounded by the target sphere of $h^\pm$.

#### Proof

First suppose $(\mathfrak {f}^\mathfrak {p},\mathfrak {t})$ to be a flat front in a hyperbolic space described by a point sphere complex $\mathfrak {p}$ and a space form vector $\mathfrak {q}$ with $\langle {\mathfrak {q},\mathfrak {q}}\rangle =1$. Using homogeneous coordinate vectors as above, $\mathfrak {s}^\pm =\mathfrak {f}^\mathfrak {p}\pm \mathfrak {t}$ and $\mathfrak {l}^\pm =\mathfrak {q}\mp \mathfrak {p}$ for the enveloped isothermic sphere congruences $s^\pm$ and their linear sphere complexes, respectively, we obtain

$$\begin{aligned} \mathfrak {h}^\pm = \pm ( \langle {\mathfrak {p},\mathfrak {l}^\pm }\rangle \mathfrak {s}^\pm - \langle {\mathfrak {p},\mathfrak {s}^\pm }\rangle \mathfrak {l}^\pm ) = \mathfrak {s}^\pm + \mathfrak {l}^\pm = (\mathfrak {f}^\mathfrak {p}+\mathfrak {q}) \pm (\mathfrak {t}-\mathfrak {p}) \end{aligned}$$

as (homogeneous coordinates of the) hyperbolic Gauss maps of the front $(\mathfrak {f}^\mathfrak {p},\mathfrak {t})$. Note that $\mathfrak {h}^\pm \perp \mathfrak {p},\mathfrak {q}$, that is, they are point sphere maps taking values in the sphere $\mathfrak {q}$ that defines the infinity boundary of the ambient hyperbolic space. As $\mathfrak {h}^\pm$ are just constant offsets of $\mathfrak {s}^\pm$, they share cross-ratios with the enveloped isothermic sphere congruences, hence are also isothermic (cf Cor 30), and form a pair of Königs dual lifts, hence yield a totally umbilic Darboux pair $(h^+,h^-)$ (see [8, Def 4.4, Thm 3.26]). Note that $\langle {\mathfrak {h}^+,\mathfrak {h}^-}\rangle =-2\ne 0$, showing that the obtained Darboux pair is non-isotropic.

Next we reverse the above construction: thus let $(h^+,h^-)$ denote a discrete Darboux pair of point spheres that take values in a 2-sphere, that is, $h^\pm \perp \mathfrak {p},\mathfrak {q}$, where $\mathfrak {p}$ denotes the point sphere complex and $\mathfrak {q}\in {\mathbb {R}}^{4,2}$ with $\langle {\mathfrak {q},\mathfrak {q}}\rangle =1$ defines a Möbius geometric sphere. Then $\mathfrak {l}^\pm :=\mathfrak {q}\mp \mathfrak {p}$ yield the two (Lie geometric) oriented spheres of this (Möbius geometric) sphere. By [8, Def 4.4, Thm 3.26] we may choose Königs dual lifts $\mathfrak {h}^\pm$ of $h^\pm$; as $\langle {\mathfrak {h}^\pm ,\mathfrak {h}^\pm }\rangle \equiv 0$ we infer (as usual) that

$$\begin{aligned} \mathfrak {h}^\pm _i + \mathfrak {h}^\pm _j \perp \mathfrak {h}^\pm _i - \mathfrak {h}^\pm _j \parallel \mathfrak {h}^\mp _i - \mathfrak {h}^\mp _j, \;\; \text{hence} \;\; \langle {\mathfrak {h}^+,\mathfrak {h}^-}\rangle \equiv -2 \end{aligned}$$

without loss of generality, after a possible (constant) rescaling of $\mathfrak {h}^+$ or $\mathfrak {h}^-$. Now observe that

$$\begin{aligned} \mathfrak {s}^\pm : = e^{\pm \rho }\mathfrak {h}^\pm + \mathfrak {l}^\pm\;\; \text{for}\;\; \rho \in {\mathbb {R}} \end{aligned}$$

yield Königs dual lifts of an isotropic Darboux pair $(s^+,s^-)$ of isothermic sphere congruences (see [8, Def 4.4]): in particular, the Legendre map $f:=s^+\oplus s^-$ projects to a flat front $(\mathfrak {f}^\mathfrak {p},\mathfrak {t})$ in the hyperbolic space(s) given by $\mathfrak {p}$ and $\mathfrak {q}$.

Finally note that, by Theorem 25, the cyclic circle congruence associated with the Darboux pair $(h^+,h^-)$ is given by

$$\begin{aligned} \Gamma = \gamma \times \gamma ^\perp : {\mathcal {V}}\rightarrow G_{(2,1)}^{{\mathcal {P}}} \times G_{(2,1)}\;\; \text{with} \;\; \gamma _i : = \text {span}\{ { \mathfrak {h}^+_i,\mathfrak {h}^-_i,\mathfrak {q} }\} . \end{aligned}$$

Clearly, $\mathfrak {s}^\pm _i\pm \mathfrak {p}\in \gamma _i$ at every vertex $i\in {\mathcal {V}}$; consequently, *f* is an orthogonal net of this cyclic circle congruence for every $\rho \in {\mathbb {R}}$, by Fact 3. $\square$

We note that the Ribaucour sphere congruence enveloped by the discrete Darboux pair $(h^+,h^-)$, considered in Theorem 31, is highly degenerate: it is the constant sphere $l^+$ or $l^-$ representing the infinity boundary of hyperbolic space. Hence the description (10) of the linear sphere complexes that subsequently induce the flat connection for the circle congruence fails. However, in this particular situation the Königs dual lifts of the Darboux pair give rise to the sought-after linear sphere complexes by

$$\begin{aligned} a_{ij} = \text {span}\{ { \mathfrak {h}^+_i-\mathfrak {h}^+_j }\} = \text {span}\{ { \mathfrak {h}^-_i-\mathfrak {h}^-_j }\} . \end{aligned}$$

These then induce the M-Lie inversions that provide the flat connection for $\Gamma$ and interchange the point spheres of the orthogonal nets on adjacent circles of the congruence.

### Discrete orthogonal systems with Dupin cyclides as coordinate surfaces

Here we investigate discrete cyclic circle congruences that stem from a discrete Ribaucour pair consisting of a discrete Dupin cyclide and a totally umbilic surface. We shall prove that these special circle congruences yield discrete cyclic systems where all coordinate surfaces are discrete Dupin cyclides.

An analogous result in the smooth case can be found in [18, 20].

Thus, suppose that $f:{\mathcal {V}} \rightarrow {\mathcal {Z}}$ is a discrete Dupin cyclide in the sense of [23], that is, a discrete channel surface with respect to both coordinate directions. In particular, any space form projection yields a circular net with circular curvature lines, so that the corresponding curvature spheres are constant along them.

Furthermore, we consider a totally umbilic discrete Ribaucour transform *u* of *f* with point spheres on the constant sphere $n \in {\mathbb {P}}({\mathcal {L}})$. For genericity, we will assume that *n* is not in oriented contact with the Dupin cyclide, that is, $n\not \subset f_i$ for all $i\in {\mathcal {V}}$.

The discrete Ribaucour sphere congruence *r* enveloped by (*f*, *u*) is then provided by the spheres in the contact elements of *f* that lie in the linear sphere complex ${\mathbb {P}}({\mathcal {L}}\cap n^\perp )$. Hence, *u* can be expressed in terms of its (constant) curvature sphere *n* and the enveloped Ribaucour sphere congruence *r* by $u_i=\text {span}\{ {\mathfrak {n},\mathfrak {r}_i}\}$ (cf [10, 30]).

To avoid useless case analyses, a totally umbilic discrete Legendre map with two families of circular curvature lines will also be called a discrete Dupin cyclide.

#### Theorem 32

Let *f* be a discrete Dupin cyclide and *u* a totally umbilic Ribaucour transform of *f*. Then the orthogonal surfaces of the discrete cyclic circle congruence associated with the Ribaucour pair (*f*, *u*) are discrete Dupin cyclides.

Furthermore, a suitable choice of contact elements for the orthogonal surfaces yields a discrete cyclic system so that all coordinate surfaces are discrete Dupin cyclides.

**Fig. 5:**
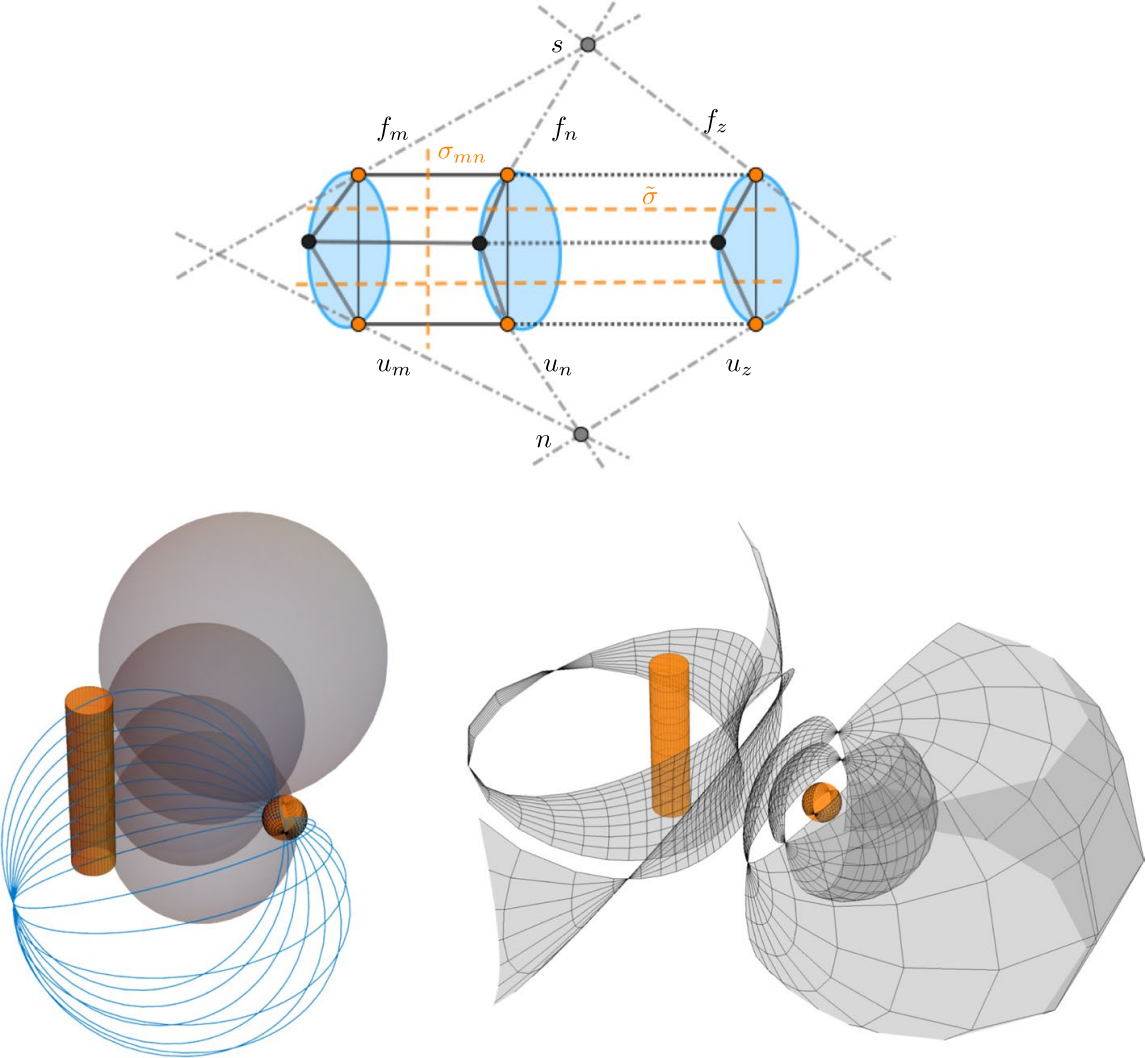
A discrete triply orthogonal system associated with the Ribaucour pair of a discrete cylinder and a totally umbilic spherical transform (orange). Equipped with suitable contact elements, all coordinate surfaces of the associated discrete cyclic system become discrete Dupin cyclides (colour figure online)

#### Proof

Let $(f,u): {\mathcal {V}}\rightarrow {\mathcal {Z}} \times {\mathcal {Z}}$ be a discrete Ribaucour pair as in the assumption. For a proof, we pursue the following line of arguments:

- along each coordinate line of the given Dupin cyclide *f*, the spheres of the enveloped Ribaucour sphere congruence are curvature spheres of a constant Dupin cyclide and, therefore, the flat connection for the associated cyclic circle congruence is of a special type (cf Cor 24 and Thm 25);
- any underlying point sphere map of a discrete cyclic system, obtained from a sampling of an initial circle, has a special property; namely, its “vertical coordinate surfaces” are multi-circular nets [6];
- hence the M-Lie inversions that relate adjacent contact elements of the associated cyclic systems are constant along each coordinate ribbon of these “vertical coordinate surfaces”;
- in particular, propagation of the contact elements of the Dupin cyclide *f* preserves circularity of the curvature lines, as well as the fact that the corresponding curvature spheres are constant along them;
- thus any orthogonal surface is a discrete Dupin cyclide and all orthogonal trajectories of the point sphere map of the associated discrete cyclic system are concircular;
- moreover, a suitable choice for the contact elements of the vertical coordinate surfaces guarantees that those are also discrete Dupin cyclides in the Lie sphere geometric sense, namely, discrete Dupin cyclides orthogonal to the Dupin cyclides formed by the Ribaucour spheres.

To begin with, we investigate the Ribaucour pair (*f*, *u*) and its Ribaucour sphere congruence along a fixed coordinate line of ${\mathcal {V}}$. Since the spheres of the contact elements of *f* along each coordinate line all lie in a 3-dimensional projective subspace of ${\mathbb {P}}({\mathbb {R}}^{4,2})$, the spheres of the enveloped Ribaucour congruence along each coordinate line are curvature spheres of another (constant) Dupin cyclide (see also [30]).

Since *f* is a discrete Dupin cyclide, along this coordinate line, all contact elements $F:=\{f_m, f_n, \cdots , f_z \}$ share a common curvature sphere; we denote this sphere by *s*. Furthermore, all contact elements $U:=\{u_m, u_n, \ldots , u_z \}$ intersect in the constant sphere *n* (for a schematic see Fig. 5*top*). Thus, these two families of contact elements provide two curvature lines on the Dupin cyclide obtained by the spheres $R:=\{r_m, r_n, \ldots , r_z\}$ of the enveloped Ribaucour sphere congruence along this coordinate line.

Therefore, additionally to the M-Lie inversions that provide the flat connection for the Ribaucour pair (cf Cor 24), we obtain further M-Lie inversions: let $(f_m, u_m)$ and $(f_z, u_z)$ be two arbitrary pairs of contact elements, then the four corresponding point spheres are concircular. Hence, there exists an M-Lie inversion $\sigma _{mz}$ so that

12 $$\begin{aligned} \sigma _{mz}(f_z)=f_m, \ \sigma _{mz}(u_z)=u_m, \ (\text {and } \sigma _{mz}(r_z)=r_m). \end{aligned}$$

Thus, this M-Lie inversion $\sigma _{mz}$ also exchanges the circles $\Gamma _m$ and $\Gamma _z$ of the orthogonal cyclic circle congruence $\Gamma$ associated with the Ribaucour pair (see Thm 25); hence, also the point spheres of its other orthogonal surfaces.

Next we investigate the underlying point sphere map of a “vertical coordinate surface” of the associated cyclic system: using the above M-Lie inversions this is given by a sampling of an initial circle, say $\Gamma _m$. We aim to see that this point sphere map is multi-circular in the sense of [6], that is, every coordinate quadrilateral is circular, not just every elementary coordinate quadrilateral.

We recall from [6, 30] that multi-circular point sphere nets may be characterized by the existence of M-Lie inversions that interchange corresponding point spheres of any two coordinate lines in one family, as those of (12) do. Thus we obtain multi-circularity of the point sphere net; and, by symmetry, similar M-Lie inversions ${\tilde{\sigma }}_{ij}$ that interchange the point spheres of coordinate lines in the other family (as illustrated in Fig. 5*top*).

For corresponding coordinate lines of two adjacent orthogonal surfaces of the cyclic circle congruence, the (constant) M-Lie inversion ${\tilde{\sigma }}$ that arises from the multi-circularity of the vertical surface may be used to transport contact elements: it clearly maps a curvature sphere that is constant along the coordinate line on one orthogonal surface to an alike curvature sphere of the other. Hence all orthogonal surfaces are, with *f*, Dupin cyclides.

Furthermore, we learn that the point spheres along any coordinate line of the underlying point sphere map of the discrete cyclic system are circular.

Finally, to obtain a discrete cyclic system with discrete Dupin cyclides as coordinate surfaces, it remains to equip the vertical coordinate surfaces of the underlying point sphere map with suitable contact elements, that is, to complement each contact element of an orthogonal surface of the cyclic circle congruence by two mutually orthogonal contact elements that are tangent to the corresponding circle of the congruence.

To do so, we fix one initial contact element $f_0$ of the Dupin cyclide *f* and consider the two (circular) coordinate lines of the underlying point sphere map of *f* that pass through the point sphere $p_0\subset f_0$: each is contained in precisely one (unoriented, Möbius geometric) sphere that is tangent to the circle $\Gamma _0$ or, equivalently, that intersects the Dupin cyclide *f* orthogonally along the given curvature line. By construction, these two spheres are orthogonal, and choosing an orientation for each of them yields suitable contact elements.

Further observe that the sphere constructed from one (circular) curvature line of *f* is invariant under the corresponding M-Lie sphere transformations $\sigma _{mn}$ along that curvature line. Consequently, propagating the just constructed contact elements at $p_0$ yields a discrete cyclic system that consists of Dupin cyclides in the Lie geometric sense. $\square$

We remark that this construction can be generalized to Ribaucour pairs of two Dupin cyclides, which also lead to associated cyclic systems with Dupin cyclides as coordinate surfaces. Note that this yields a “totally cyclic system,” where each coordinate direction provides a cyclic circle congruence. Details regarding this construction in the smooth case can be found in [33]. A suitable sampling then yields a construction in the discrete case.
